# Single-Source Deposition of Mixed-Metal Oxide Films Containing Zirconium and 3d Transition Metals for (Photo)electrocatalytic Water Oxidation

**DOI:** 10.1021/acs.inorgchem.2c00403

**Published:** 2022-04-12

**Authors:** Victor Riesgo-Gonzalez, Subhajit Bhattacharjee, Xinsheng Dong, David S. Hall, Virgil Andrei, Andrew D. Bond, Clare P. Grey, Erwin Reisner, Dominic S. Wright

**Affiliations:** † Yusuf Hamied Department of Chemistry, University of Cambridge, Lensfield Road, Cambridge CB2 1EW, United Kingdom; ‡ College of Chemical Engineering, Nanjing Forestry University, Nanjing 210037, Jiangsu, China; § The Faraday Institution, Quad One, Harwell Science and Innovation Campus, Didcot OX11 0RA, United Kingdom

## Abstract

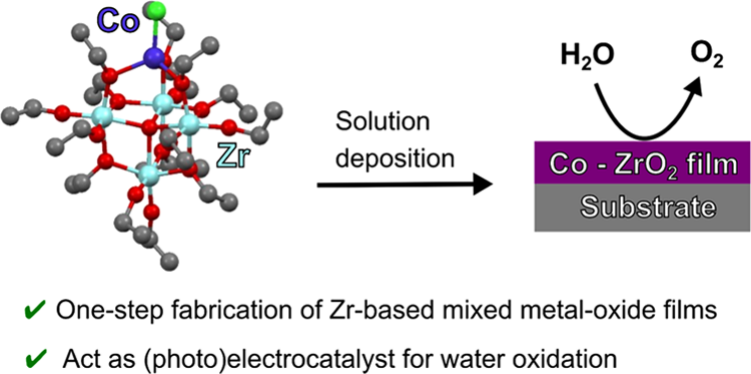

The fabrication of mixed-metal oxide films holds promise for the development of practical photoelectrochemical catalyst coatings but currently presents challenges in terms of homogeneity, cost, and scalability. We report a straightforward and versatile approach to produce catalytically active zirconium-based films for electrochemical and photoelectrochemical water oxidation. The mixed-metal oxide catalyst films are derived from novel single-source precursor oxide cage compounds containing Zr with first-row transition metals such as Co, Fe, and Cu. The Zr-based film doped with Co on fluorine-doped tin oxide (FTO)-coated glass exhibits the highest electrocatalytic O_2_ evolution performance in an alkaline medium and an operational stability above 18 h. The deposition of this film onto a BiVO_4_ photoanode significantly enhances its photoelectrochemical activity toward solar water oxidation, lowering the onset potential by 0.12–0.21 V vs reversible hydrogen electrode (RHE) and improving the maximum photocurrent density by ∼50% to 2.41 mA cm^–2^ for the CoZr-coated BiVO_4_ photoanodes compared to that for bare BiVO_4_.

## Abstract 2

New mixed-metal zirconium-oxo cages containing Co, Fe, and Cu are synthesized and used as single-source precursors for the fabrication of (photo)electrocatalytic transition-metal metal-doped zirconium oxide films. A mixed-metal Co complex showed the greatest activity in water oxidation when coated onto FTO, which was further improved when integrated into a BiVO_4_-coated FTO film. The highest photocurrent density of 2.42 mA·cm^−2^ (at 1.23 V vs RHE), with an onset potential of 0.21 V (vs RHE), was observed for this FTO|BiVO_4_|CoZr system. This is the first application of mixed-metal zirconium-based single-source precursors in this area.

## 1 Introduction

Hydrogen gas is widely used in industry for petroleum refining, float glass production, and the synthesis of fertilizers. In the future, it is also considered as a fuel for energy storage, transport, and more sustainable manufacturing.^1^ Therefore, it is likely to hold an essential role in achieving a zero-carbon economy, but we need to develop clean and efficient ways to produce it at scale and at a low cost. Water electrolysis and photoelectrochemical (PEC) water splitting are promising technologies toward this end^2−5^ but are still limited by the sluggish kinetics of the four-electron water oxidation reaction when using inexpensive catalysts.^6−10^ The costs of these catalysts can be reduced by replacing noble metals with more earth-abundant elements, such as first-row transition metal (TM)s. However, this leads to challenges such as low efficiencies and fast degradation. Although significant progress in the fabrication of noble-metal free oxygen evolution reaction (OER) catalysts has been achieved,^11−18^ the challenges of high overpotential, low stability, and complex electrode fabrication techniques still persist. It is therefore desirable to develop noble-metal free OER catalysts that show enhanced performance as well as methods for their production at scale.

Furthermore, given that increasing use of renewable energy sources such as sunlight is a key step toward sustainability, PEC catalysts for light-driven water oxidation are being developed.^4^ Toward this end, n-type semiconducting materials with an optimum band-gap for water oxidation are commonly used.^19,20^ The BiVO_4_ anode is particularly promising as it allows unassisted tandem PEC water oxidation due to its early onset potential.^21,22^ However, it suffers from rapid charge recombination that hampers its photoactivity.^23,24^ The deposition of a cocatalyst layer on top of the BiVO_4_ overcomes this problem by allowing better charge extraction, decreasing recombination losses, and increasing stability under operation.^23,25^ Integrating cocatalysts with BiVO_4_ is therefore emerging as a promising strategy to produce solar-driven water oxidation devices with improved performance.^26−28^

A major limitation in the fabrication of these composite catalysts is the available deposition techniques, namely, electrodeposition, photodeposition, and wet-chemistry routes. Electrodeposition^29,30^ suffers from sensitive voltage protocols, scalability issues, and the inability for the direct deposition on semiconductors. Photodeposition^31^ requires UV irradiation, and wet chemistry^32^ is usually followed by high-temperature treatments to obtain the desired material. Furthermore, both photodeposition and wet-chemistry approaches typically require the use of excess catalyst reagents. An easily scalable, simpler route toward the fabrication of these films is therefore urgently needed.

In this context, the use of mixed-metal single-source precursors (SSPs) presents a unique opportunity for the facile deposition of metal oxides onto a variety of substrates, including photoactive semiconducting electrodes that show a high catalytic activity toward water oxidation.^33,34^ The simple approach of drop-casting or spin-coating the precursor solutions onto the substrate affords the formation of the catalytic film providing a low-cost and easily scalable approach to the fabrication of catalysts for the water-splitting reaction.^34−36^ This deposition process can be mediated by the alkoxy groups at the periphery of the precursor molecules. These groups hydrolyze in the presence of ambient air providing the means by which thin films of material can be deposited directly from the solution. This enables the single-step deposition of a cocatalyst layer over semiconductors such as Si, WO_3_, and BiVO_4_. The deposited mixed-metal oxide films not only furnish efficient electrocatalysts involving active first-row TMs but can also protect the semiconductor electrode from corrosion. Furthermore, the homogeneous distribution of the active dopants in the inert host material can potentially improve the metal-atom utilization by increasing the number of available active sites.^37^

Consequently, we have explored the applications of polyoxotitanium cages as SSPs for the deposition of TM-doped titania, both in regard to their use in pollution control and water splitting.^28,34−36,38^ Zr-based SSPs, on the other hand, are virtually unexplored for the fabrication of mixed-metal oxide coatings for (photo)electrocatalysis. This is surprising given that Zr is nontoxic, forms a moisture-stable oxide, and is the 11th most abundant metal in the earth’s crust. Zirconia (ZrO_2_)-based systems are therefore attractive hosts for redox-active first-row TMs. Furthermore, the high charge-to-size ratio of Zr^4+^ gives it strong Lewis acid character,^39−41^ which may help to stabilize reaction intermediates during catalysis, similar to the way that Ca^2+^ acts as a Lewis acid to stabilize water oxidation intermediates in the heterometallic CaMn_4_ clusters in photosystem II.^42−46^

Although the fabrication of 3d-TM-doped zirconia catalysts using SSPs has not been explored previously, a few reports of Zr-containing electrocatalysts incorporating 3d-TMs exist. For example, Zr borides and phosphides containing 3d-TMs have been prepared and tested as water-splitting catalysts in the context of intercalation of active species that act as confined catalytic centers.^47−51^ Oxides such as CoFe_2_O_4_ have also been doped with Zr accessing different morphologies with increased surface area.^52^ These methods for the fabrication of Zr-based mixed-metal films usually require annealing at high temperatures for the solid-state reaction of the precursors^51,53^ or multistep synthetic routes for the incorporation of the dopant.^50,54^ Moreover, MOFs containing a variety of transition metals acting as active sites have also been employed in electrocatalysts.^55,56^ However, these usually present their own challenges, such as poor conductivity and stability, and are difficult to scale up. In contrast to all of these approaches, film deposition from the solution using SSPs allows the room-temperature deposition of mixed-metal oxide films in a one-step process using scalable methods such as drop-casting or spin-coating.^33^

With this background in mind, we have developed three different Zr-based mixed-metal SSPs and investigated their activity toward electrochemical water oxidation and as cocatalysts for PEC water oxidation. The precursors are Zr-based cage compounds incorporating first-row TMs in their structures. The drop-casting of the precursors affords the first examples of room-temperature deposition of TM-doped amorphous ZrO_2_ films (abbreviated as MZr where M = Co, Fe, and Cu). The CoZr system (**1**) on a FTO-coated glass substrate (FTO|CoZr) showed the highest electrocatalytic activity out of the three catalysts tested in an alkaline medium and improved the photoactivity of BiVO_4_ under neutral pH substantially. This work demonstrates the suitability of the single-source precursor approach for the facile synthesis of technologically relevant complex metal oxides. Furthermore, we highlight the role of the TM cocatalyst in the design of efficient water-splitting catalyst films.

## 2 Experimental Methods

### 2.1 Precursor Reagents

Zirconium(IV) ethoxide (Zr(OEt)_4_, Sigma-Aldrich, 98%), iron(II) chloride (FeCl_2_, Sigma-Aldrich, 98%), cobalt(II) chloride (CoCl_2_, Sigma-Aldrich, ≥97%), copper(II) chloride (CuCl_2_, Sigma-Aldrich, ≥97%), dimethyl sulfoxide (DMSO, Alfa Aesar, ≥99%), anhydrous ethanol (EtOH, Sigma-Aldrich), dry tetrahydrofuran (THF, Sigma-Aldrich), potassium hydroxide (KOH, Sigma-Aldrich, semiconductor grade, ≥99%), Nafion 117 solution, bismuth nitrate pentahydrate (Bi(NO)_3_·5H_2_O, Sigma-Aldrich, 98%), sodium iodide (NaI, laboratory reagent grade, Fischer Scientific), *p*-benzoquinone (≥ 98%, Sigma-Aldrich), vanadyl acetylacetonate (≥97%, Fluka) were used.

### 2.2 Precursor Synthesis

Strict inert-atmospheric conditions were used throughout all of the syntheses of **1–3**. Anhydrous chloride salts M^II^Cl_2_ with M = Co, Cu, Fe, and ZrOEt_4_ (98%) were acquired from Aldrich chemical company. EtOH was distilled over Mg turnings and tetrahydrofuran (THF) over sodium/benzophenone under a nitrogen atmosphere. Teflon-lined (23 mL capacity) autoclaves (model 4749, Parr) were used for all experiments. Autoclaves were heated using a Binder ED53 53 L oven with natural convection. The reactions were loaded with the solvents and reagents inside a Saffron Scientific (type β) glovebox, equipped with a closed-loop recirculation system for the removal of moisture and oxygen (operating at ca. 0.1–0.5 ppm O_2_). Storage of the products and analytical and spectroscopic samples were prepared inside the glovebox.

#### 2.2.1 Synthesis of [Zr_4_O(EtO)_15_Co^II^Cl] (**1**)

Zirconium(IV) ethoxide (1.132 g, 4.18 mmol), cobalt(II) chloride (102 mg, 0.78 mmol), and anhydrous EtOH (5 mL, 85.7 mmol) were placed in a Teflon-lined autoclave and heated at 100 °C for 1 day. After cooling down to room temperature, the EtOH was evaporated, and the residue was crystallized from 5 mL dry THF at −14 °C to produce purple crystals of **1**, which were dried under vacuum (0.53 g, 27% yield on the basis of CoCl_2_ supplied). Elemental analysis found C 29.9, H 5.7, calc. for **1** C 31.3, H 6.5.

#### 2.2.2 Synthesis of [Zr_4_(O)_2_(EtO)_16_Fe_2_^III^Cl_2_] (**2**)

Zirconium(IV) ethoxide (1.132 g, 4.18 mmol), iron(II) chloride (99 mg, 0.78 mmol), and anhydrous EtOH (5 mL, 85.7 mmol) were placed in a Teflon-lined autoclave and heated at 100 °C for 1 day. After cooling to room temperature, the EtOH was evaporated, and the residue was crystallized from 5 mL of dry THF at −14 °C to produce pink crystals of the solvate **2**.2THF, which were dried under vacuum (0.13 g, 11% yield on the basis of providing FeCl_2_). Elemental analysis found C 29.0, H 5.9, calc. for **2** C 29.5, H 6.1. The elemental analysis shows that the THF present in the crystalline lattice is completely removed under vacuum, which also leads to the loss of crystallinity.

#### 2.2.3 Synthesis of [Zr_4_(O)_2_(EtO)_18_Cu_4_^II^Cl_4_] (**3**)

Zirconium(IV) ethoxide (1.132 g, 4.18 mmol), copper(II) chloride (105 mg, 0.78 mmol), and anhydrous EtOH (5 mL, 85.7 mmol) were placed in a Teflon-lined autoclave and heated at 100 °C for 1 day. After cooling to room temperature, the EtOH was evaporated, and the residue was crystallized in 5 mL of dry THF at −14 °C to produce blue crystals of **3**.EtOH (0.41 g, 62% yield on the basis of CuCl_2_ provided). Elemental analysis found C 25.7, H 5.3, calc. for **3** C 26.9, H 5.6. The elemental analysis shows that the EtOH present in the crystalline lattice is completely removed under vacuum, which also leads to the loss of crystallinity.

### 2.3 IR Spectroscopy Measurements

IR spectra were recorded as Nujol mulls using a PerkinElmer 1000 spectrophotometer with a universal ATR using NaCl windows.

### 2.4 UV–vis Spectroscopy Measurements

The spectra were measured using a Varian Cary 50 spectrophotometer at 25 °C using quartz crystal cells. UV-diffuse-reflectance spectroscopy (UV-DRS) was measured using a Harrick Scientific Video Barrelino probe.

### 2.5 Elemental Analysis

Elemental CHN analysis was obtained using an Exeter Analytical, Inc. CE-440 elemental analyzer with a combustion temperature of 975 °C.

### 2.6 ICP-OES Measurements

An iCAP 7400 Series ICP spectrometer from Thermo Fisher Scientific was used to detect the Co, Cu, and Fe content in the film samples. Samples for analysis were dissolved in 2% HNO_3_ to an estimated 1 ppm (mg L^–1^) concentration (5–10 mL total volume).

### 2.7 Single-Crystal X-ray Diffraction

All single-crystal X-ray data were collected at 180(2) K using a Nonius KappaCCD diffractometer equipped with Mo Kα radiation (λ = 0.7107 Å). Crystallographic data and refinement details are included in the Supporting Information. Crystallographic data in CIF format have been deposited with the Cambridge Crystallographic Data Centre (CCDC 2011723–2011725).

### 2.8 Fabrication of Electrocatalysts

FTO-coated glass substrates were thoroughly cleaned by sonication in MilliQ water, acetone, and isopropanol (20 min in each), followed by UV-ozone treatment for 15 min. To obtain a uniform deposition, 20 μL of the saturated catalyst solution (concentration 0.26 mol L^–1^) was carefully pipetted onto a clean preheated (40 °C) FTO-coated glass substrate (effective area 1 cm^2^) and dried for 15 min under ambient conditions. This process was repeated two more times, for a total of 60 μL of solution. Thereafter, 20 μL of a 1:1 Nafion/EtOH mixture was uniformly pipetted over the catalyst layer and dried overnight under ambient conditions. The amorphous nature of the film was confirmed by powder XRD, which did not show any peaks between 5 and 80 2-theta degrees. The same drop-casting procedure was followed when preparing the carbon-fiber paper, graphite paper, and ITO-coated glass substrates.

### 2.9 Fabrication of FTO|BiVO_4_|CoZr Photoanodes

BiVO_4_ photoanodes were prepared according to previous reports.^21,35^ To employ CoZr as a cocatalyst for solar-driven water oxidation, the saturated solution of the CoZr precursor was first diluted using a 1:1 THF/EtOH mixture at different concentrations (0.13, 0.052, 0.026, 0.013, 0.0065 mol L^–1^). Next, 20 μL of the diluted sample was spin-coated on top of the BiVO_4_ films at 2000 rpm for 10 s, 2000 rpm s^–1^ acceleration. The dilution and spin-coating were necessary to yield a uniform thin-layer deposition and avoid light blockage leading to poor light absorption by BiVO_4_. Twenty microliters of a 1:1 Nafion/EtOH mixture was then spin-coated over the FTO|BiVO_4_|CoZr photoanode and dried overnight under ambient conditions.

### 2.10 Materials Characterization

Scanning electron microscopy/energy-dispersive X-ray spectroscopy (SEM/EDS) were obtained using a TESCAN MIRA3 FEG-SEM (FEG = field emission gun). SEM images were acquired at 5 or 15 kV acceleration voltages, and EDS spectra were acquired at a 30 kV acceleration voltage. X-ray photoelectron spectroscopy (XPS) was measured on a Thermo Fisher Scientific EscaLab 250 Xi equipped with a monochromatic Al Kα X-ray source (hν = 1486.68 eV) and a spot size of 500 μm × 500 μm. Survey spectra were measured at a pass energy of 100 eV, and high-resolution spectra were measured at a pass energy of 20 eV. On account of closures due to the pandemic, the free-standing CuZr was measured at another facility, using a Thermo Fisher Scientific K-Alpha XPS system, also equipped with a monochromatic Al Kα X-ray source (hν = 1486.69 eV). The survey spectrum was measured at a pass energy of 160 eV, and the high-resolution spectra were measured at a pass energy of 20 eV. In all cases, spectra were charge-corrected by setting the adventitious carbon alkyl (C–H, C–C) peak to 284.8 eV (Figures S10 and S11). A detailed description of the calibration method and additional spectra are provided as the Supporting Information and follow conventional practices.^57^

### 2.11 Electrochemical and Photoelectrochemical Measurements

The electrochemical measurements were performed using an Ivium compactstat electrochemical analyzer and a two-compartment cell separated by a Selemion anion-exchange membrane. The electrochemical measurements were performed in alkaline conditions; 1 M KOH_(aq)_ was used as the electrolyte solution, which was purged with N_2_ prior to the experiments. The FTO|MZr (M = Co, Fe, Cu) catalyst, Ag/AgCl_sat_, and platinum mesh were used as the working, reference, and counter electrodes, respectively. Prior to the electrochemical measurements (except 4 h chronoamperometry), the working electrode was subjected to CV scans between 0.8 and 2.0 V vs reversible hydrogen electrode (RHE) at a scan rate of 100 mV s^–1^ for 20 cycles to activate it and remove surface impurities.^58^ Linear sweep voltammetry (LSV) measurements were performed at a lower scan rate of 5 mV s^–1^ for all of the three systems to minimize the capacitive current. Unless mentioned otherwise, all of the potentials reported have been converted from Ag/AgCl_sat_ to the reversible hydrogen electrode (RHE) scale using eq 1, where pH of 1 M KOH (aq) was estimated to be 14.^59^
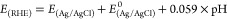
1The potential of the saturated Ag/AgCl electrode was taken as *E*_Ag/AgCl_^0^ = 0.197 *V*_NHE_. To account for the uncompensated solution resistance and other Ohmic losses, the measured potentials were IR-corrected using eq 2, where *R*_Ω_ was determined from the Bode Plots of the respective catalysts (eq 2).
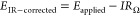
2The turnover frequencies of the catalysts were calculated using eq 3, where *I* is the current (in A m^–2^; determined at 490 mV vs RHE overpotential for our systems), *z* is the number of electrons involved in the OER process (*z* = 4), *F* is the Faraday constant (96 580 C mol^–1^), and *n* is the concentration of active catalytic sites (determined from ICP-OES; in mol m^–2^)^60^
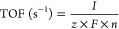
3The stability tests for each catalyst system were done using chronoamperometry by applying a constant bias of 1.6 V vs RHE for CoZr and 1.8 V vs RHE for FeZr and CuZr, for 18 h. The double-layer capacitance (*C*_dl_) was determined by performing CV scans at different scan rates in the non-faradaic region. The slope of the current vs scan rate plot yields the *C*_dl_, according to eq 4
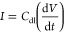
4The electrochemically active surface area (ECSA) was determined from the *C*_dl_ by dividing the *C*_dl_ by the specific capacitance *C*_s_ (*C*_s_ = 40 μF cm^–2^) known from literature.^61,62^

The PEC measurements were performed with a Newport Oriel 67005 solar light simulator with an AM 1.5G solar filter. The irradiance (i.e., flux density) was calibrated to 100 mW m^–2^ using a Newport 116-R optical power meter. Since BiVO_4_ is not stable under alkaline conditions, 0.1 M potassium borate (KBi) buffer (pH 8.5) with 0.1 M K_2_SO_4_ as a supporting electrolyte was used for the PEC measurements. Prior to the PEC measurements, the electrolyte solution was purged with N_2_ for 20 min. The CV scans (chopped, light, and dark) were run between 0.1 and 1.4 V vs RHE at a low scan rate of 10 mV s^–1^. The scans were performed under back illumination to ensure efficient light absorption.

The O_2_ quantification was conducted for the best-performing system CoZr in the anodic compartment of a gas-tight (photo)electrochemical cell using an Ocean Optics fluorescence oxygen probe (Forpor-R). A constant potential was applied for 4 h. The O_2_ baseline was also recorded before and after the chronoamperometry. The amount of oxygen in the solution was obtained using Henry’s law.

## 3 Results and Discussion

### 3.1 Synthesis and Characterization of Catalyst Precursors

We employed one-step synthetic approaches to the new complexes [{Zr_4_(μ_4_-O)(EtO)_15_}Co^II^Cl] (**1**), [{Zr_4_(μ_4_-O)_2_(EtO)_16_}(Fe^III^Cl)_2_] (**2**), and [{Zr_4_(μ_4_-O)_2_(EtO)_16_}{(Cu^II^Cl)_2_(OEt)}_2_] (**3**), in which Zr(OEt)_4_ is reacted with the corresponding first-row TM salt (MCl_2_; M = Co, Fe, Cu) in a 5:1 molar ratio in EtOH under solvothermal conditions (see Section 2). The formation of oxo-cage compounds in these reactions has been ascribed to the presence of trace water in the solvent or starting materials or to scavenging of O-atoms from the EtOH solvent (with the formation of diethyl ether) under the solvothermal conditions used.^63^ The products **1–3** were obtained as crystals after workup in 11–62% yield. Compounds **1–3** were characterized by infrared (Figure S9), solution and solid-state UV–visible spectroscopy, and elemental (C, H) analysis.

**Figure 1:**
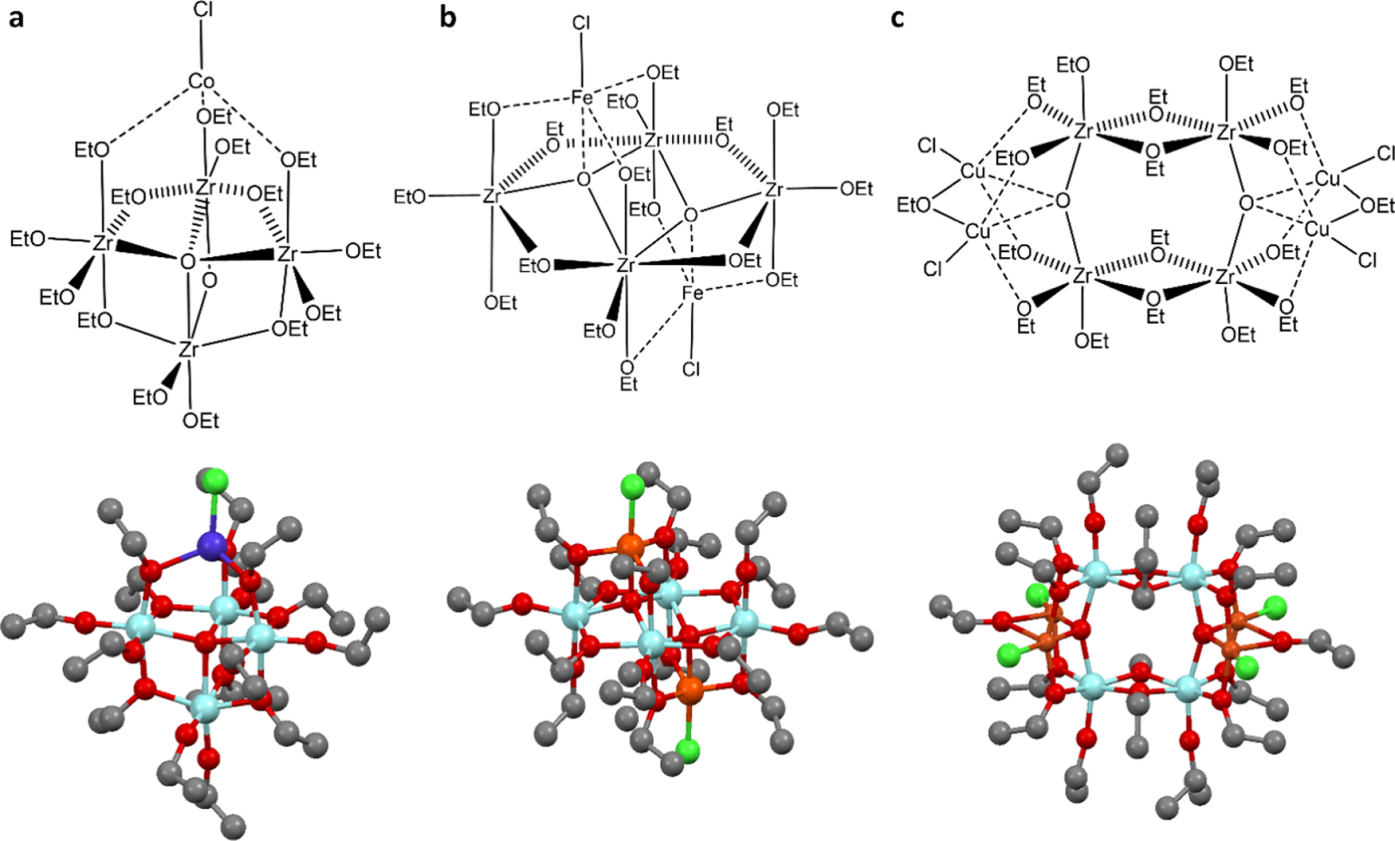
Molecular structures of SSPs. (a) [{Zr_4_(μ_4_-O)(OEt)_15_}Co^II^Cl] (**1**). H-atoms and one of the disordered CoCl sites (present in 50:50 ratio with the site shown) have been omitted for clarity. (b) [{Zr_4_(μ_4_-O)_2_(EtO)_16_}(Fe^III^Cl)_2_] (**2**). H-atoms and the lattice THF molecules have been omitted for clarity. (c) [{Zr_4_(μ_4_-O)_2_(EtO)_16_}{(Cu^II^Cl)_2_(OEt)}_2_] (**3**). H-atoms and lattice EtOH molecules have been omitted for clarity. For details of the structural refinements and selected bond lengths and angles, see Supporting Information Section 1 and Tables S1 and S2.

The single-crystal X-ray structures of **1**–**3** are shown in Figure 1, and details of the structural refinements and data collections can be found in the Supporting Information. Their compositions reveal a 4:1 ratio of Zr/Co in **1**, 4:2 ratio of Zr/Fe in **2**, and 1:1 of Zr/Cu in **3**, showing that the reaction stoichiometry has little or no bearing on the molecular ratio or structure of the complex formed. Instead, it appears that the coordination preference and ionic size of the “dopant” metal ions (Co^2+^, Fe^2+^, and Cu^2+^) have the greatest effect on structure. Previous work has led to similar conclusions regarding the structures of heterometallic polyoxotitanium complexes containing Co^2+^, Cu^2+^, and Fe^2+^.^34,38^

**Figure 2:**
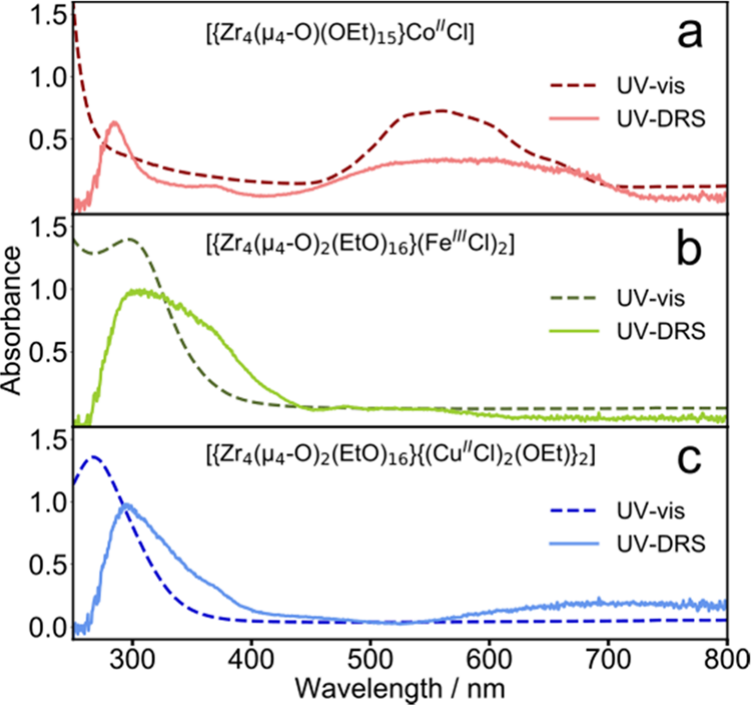
Solution UV–vis and DRS UV–vis spectra of precursors **1**–**3**. The solution UV–vis spectra were recorded in dry EtOH (conc. 0.08 mmol L^–1^). The data show the expected absorption bands for tetrahedral d^7^ and d^9^ configurations, indicating the presence of Co^2+^, and Cu^2+^, respectively. Furthermore, the lack of absorption bands in the visible region of **2** is consistent with the presence of trigonal bipyramidal Fe^3+^ with a d^5^ configuration.

Compound **1** (Figure 1a) is isostructural with the series of first-row TM complexes [{Ti_4_(μ_4_-O)(OEt)_15_}M^II^Cl] with M = Co, Cu, Fe.^64,65^ Its crystal structure is fully isomorphous with Co and Fe titanium complexes and also with the zirconium-based ZnCl complex [{Zr_4_(μ_4_-O)(EtO)_15_}Zn^II^Cl] reported previously.^66^ The complex can be regarded as being constructed from a [{Zr_4_(μ_4_-O)(OEt)_15_}]^−^ anion and a single [Co^II^Cl]^+^ fragment. The Co^2+^ ion is coordinated by three of the EtO^–^ groups of the anion together with a Cl^–^ ligand, resulting in a distorted tetrahedral metal geometry (with all of the Zr^4+^ centers having six-coordinate, distorted-octahedral geometries). Direct evidence of the presence of Co^2+^ in this arrangement is observed in the UV–visible spectrum of **1** (Figure 2a), showing three bands in the visible region (561, 600, and 658 nm), which correspond to the three allowed transitions from the ^4^A ground state to the three ^4^T states for a d^7^ tetrahedral ion.

The structures of **2** and **3** (Figure 1b,c) are unprecedented in relation to the known family of related alkoxy first-row mixed-metal polyoxozirconium cages, which generally contain Zr_2_ or Zr_3_ units.^67−70^ The crystal structure of **2** is unambiguous, but some uncertainty remains over the precise composition and structure of **3**. The X-ray data indicate that **3** consists of a [Zr_4_(μ_2_-O)_2_(EtO)_16_]^4–^ anion, which coordinates two [(CuCl)_2_(μ_2_-OEt)]^+^ units at either side of its rectangular Zr_4_ unit. This leaves an overall 2^–^ charge on the complex, with no apparent charge-balancing cation(s) within the crystal structure. Possibilities for charge balance include protonation of two of the ethoxide ligands or exchange of Cl^–^/EtO^–^ by H_2_O; these aspects are discussed in the Supporting Information. The Cu^2+^ ions have pseudotetrahedral coordination geometries, with all of the Zr^4+^ centers having distorted-octahedral environments.

The solution UV and diffuse-reflectance spectroscopy (DRS) UV–vis spectra of the solid are presented in Figure 2. DRS UV–vis allowed the observation of weak absorptions that were not seen in solution UV–vis because of the low solubility of the compounds. The absorption bands at 280–300 nm are attributed to the Zr–O cage backbone.^71^ The presence of Cu^2+^ (Figure 1c) is supported by the observation of a single visible absorption band at ca. 690 nm in the solid-state UV-DRS spectrum, which corresponds to the weak-field ^2^T → ^2^E for a d^9^ tetrahedral TM ion and in agreement with previously reported titanium-oxo cages containing Ti^4+^.^64^ The *C*_2h_-symmetric molecular structure of **2** contains a [Zr_4_(μ_3_-O)_2_(EtO)_16_]^4–^ anion with the same composition as the anion in **3** but a very different arrangement. In **2**, each of the oxo-anions bridges three of the four Zr^4+^ ions within the boundary of the rhombic Zr_4_ unit (rather than being located at two opposite edges of a rectangular metal framework). The Fe^3+^ cations have distorted trigonal bipyramidal geometries, each being coordinated by an oxo atom of the core, by three-terminal EtO^–^ groups and by a terminal Cl^–^ ligand. The absence of any absorption band in the visible region in the UV–vis spectrum of **2** is consistent with the presence of high spin d^5^ Fe^3+^ cations in a complex in which d–d electronic transitions are spin-forbidden.

### 3.2 Catalyst Characterization

**Figure 3:**
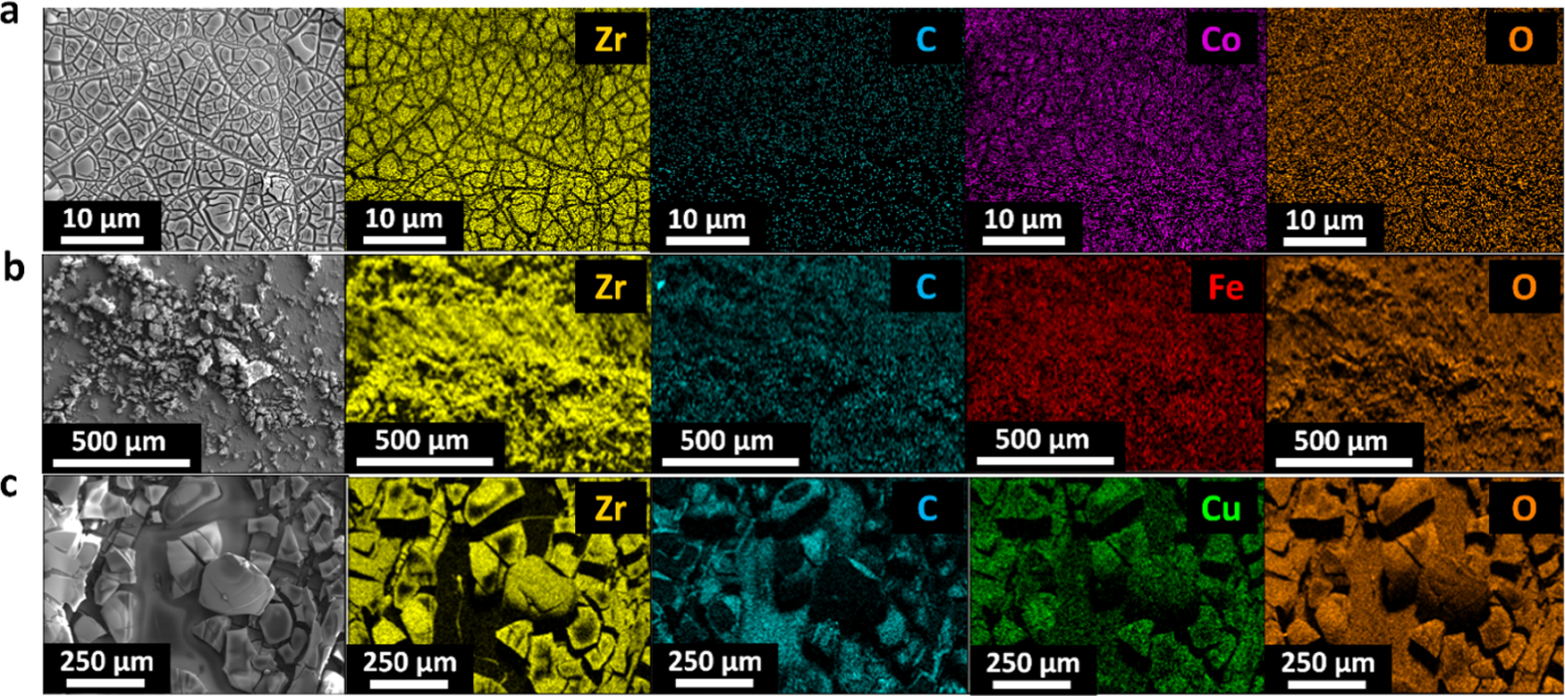
SEM images and corresponding EDS elemental mapping of the three systems: (a) FTO|CoZr, (b) FTO|FeZr, and (c) FTO|CuZr.

The electrocatalyst films were prepared by drop-casting an SSP solution (60 μL, 0.26 mol L^–1^) onto a clean, preheated (40 °C) FTO-coated glass substrate. The resulting films are designated as FTO|MZr. Scanning electron microscopy (SEM) images and energy-dispersive X-ray spectroscopy (EDS) maps presented in Figure 3 show the morphologies and elemental compositions of the samples. SEM images show that the deposition process leads to rough films on top of the substrate. The morphologies of the films vary significantly depending on the region of the sample, being composed of fragments of material that show large differences in size ranging from 1 to 250 μm for each of the electrodes. EDS maps confirm the presence of zirconium and the dopant element evenly distributed throughout the deposited layer.

**Figure 4:**
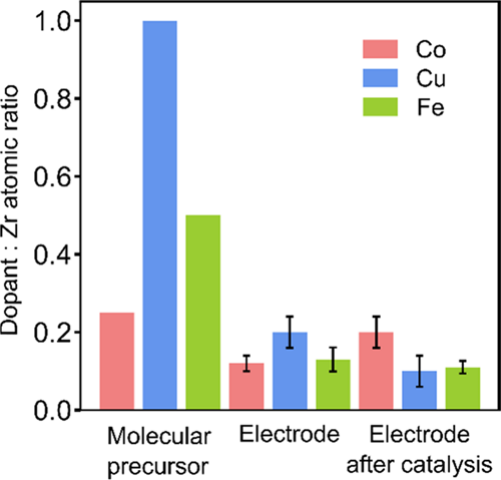
Dopant-to-zirconium atomic ratios for the molecular precursors, determined from their crystal structure, and the catalyst films deposited onto FTO glass, determined by EDS. The dopant content of the catalyst is lower but dependent on that of the precursor. The metal dissolution during operation is dependent on the combination of elements present.

To obtain quantitative information about the composition of the catalysts, multiple EDS spectra were measured for each of the samples. The atomic ratios were calculated from these data and are shown in Figure 4. The similar levels of dopant content detected on different points of the sample confirm that our approach allows the deposition of doped-zirconia films that are homogeneous in composition (see Tables S3–S8). The catalysts have lower dopant (Co, Fe, Cu) content than the precursor molecules used as SSPs, suggesting that a fraction of the dopant remains in the solution during the deposition process and is not incorporated into the materials. Nevertheless, there is a significant amount of dopant incorporated into the materials, and there is a correlation between dopant levels in the precursor and the catalyst, with precursors containing higher dopant content leading to a larger amount of dopant in the catalyst film. The presence of first-row TMs in the catalyst film and the independence of the amount of dopant on the dopant/Zr ratio of the parent precursor was further confirmed by ICP-OES, which showed values of Co, Fe, and Cu of 0.4–0.5 mol cm^–2^ (Table S11). Finally, we studied the catalyst composition after operation in water oxidation following the same methodology to investigate the stability and possible metal dissolution. We find that, within error, there are similar levels of dopants present before and after catalysis for the three materials. For the FTO|CuZr system, *a* slight decrease in Cu content after catalysis is observed, while for the FTO|FeZr system, the Fe content remains the same, within error, before and after catalysis. The EDS analysis of the FTO|CoZr system showed a slight increase in the Co/Zr ratio after catalysis. The EDS results do not support Zr dissolution as similar Zr atomic % values are detected before and after catalysis (12.2 ± 1.0 and 15.2 ± 1.8, Tables S3 and S4). Alternatively, this enrichment in surface Co might be explained by a surface reconstruction process.^72,73^

**Figure 5:**
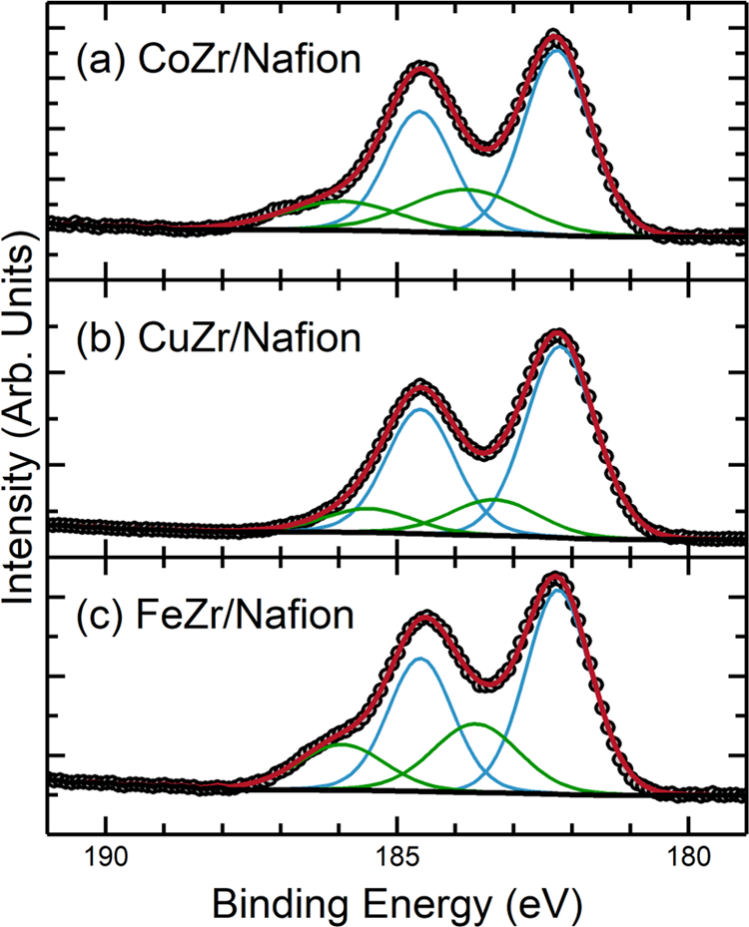
High-resolution XPS of the Zr 3d region for (a) FTO|CoZr, (b) FTO|CuZr, and (c) FTO|FeZr samples. The spectra were fitted using two spin–orbit 3d_5/2_/3d_3/2_ pairs (i.e., four peaks total).

X-ray photoelectron spectroscopy (XPS) was applied to the deposited films to investigate the chemical species present on their surfaces (Figure 5). The Zr 3d region was fitted by constraining the 3d_5/2_ and 3d_3/2_ spin–orbit pairs to have equal peak width and a fixed area ratio of 3:2. In all cases, it was found that the region fitted very well using two chemical environments (i.e., four peaks in total). The lower binding-energy 3d_5/2_ components were centered at 182.2–182.3 eV for all three films, and the spin–orbit splitting was in the region 2.37–2.39 eV, consistent with reported values for the Zr(IV) oxide, ZrO_2_ (182.8 ± 0.6 and ∼2.43 eV, respectively^74^). The higher binding-energy (BE) 3d_5/2_ component had more variability between samples and was centered at 183.81, 183.15, and 183.59 eV for the FTO|CoZr, FTO|CuZr, and FTO|FeZr samples, respectively. The higher BE peaks are above the binding-energy range expected for *a* metal alloy (e.g., Zr–Co alloys and alloy films, BE ≅ 178.9 eV).^75^ Thus, they are attributed to mixed-metal oxides, supported by the presence of the respective TM counterparts (Co 2p, Cu 2p, and Fe 2p spectra are shown in Figure S15). These results are consistent with the findings from EDS, i.e., that the metal ratios in the deposited films are different from those of the precursors. It is important to note that the analysis depth of XPS is significantly lower (order of a few nanometers) than EDS. The fitted XPS peak parameters are summarized in Table S10.

**Figure 6:**
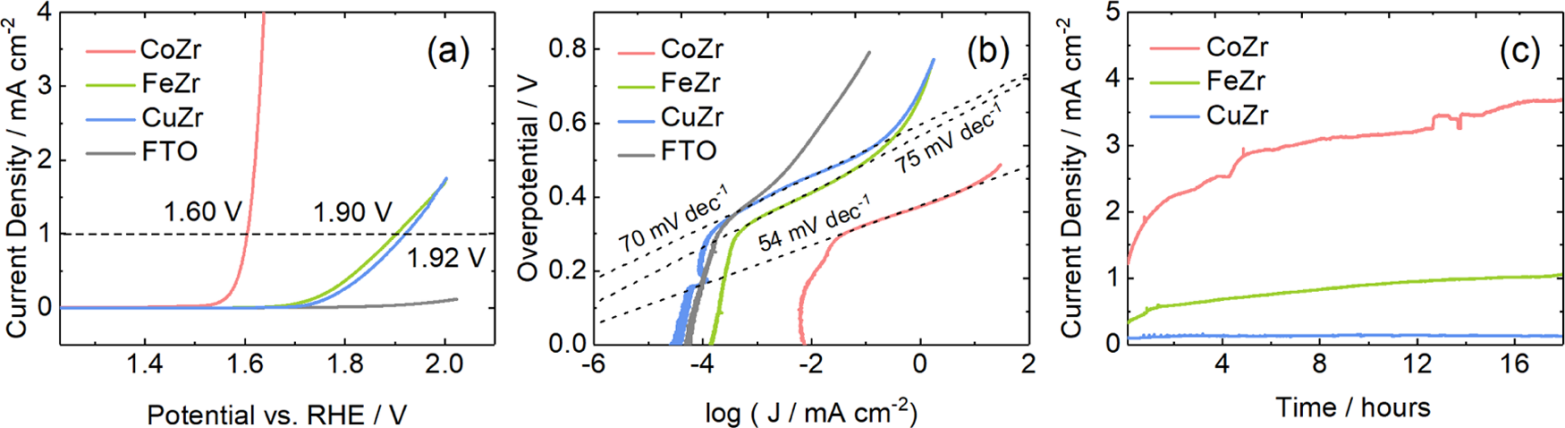
(a) Linear sweep voltammetry (LSV) was measured on the untreated FTO glass (gray) and on the FTO|CoZr (red), FTO|FeZr (green), and FTO|CuZr (blue) coatings (currents normalized to geometric surface area of the electrodes, i.e., 1 cm^2^; scan rate 5 mV s^–1^) and the corresponding (b) Tafel plots and linear fittings. (c) Eighteen hour chronoamperometric (CA) tests were performed at a constant potential of 1.8 V for FTO|CuZr and FTO|FeZr and 1.6 V for FTO|CoZr owing to the earlier onset of oxygen evolution of the later. Measurements were performed in degassed 1 M KOH_(aq)_ solutions at room temperature.

The deposited films were then tested for electrocatalytic activity toward the OER; following a series of CVs performed to clean the materials, LSV measurements were taken in the direction of increasing potential (Figure 6). The CuZr film initially shows very similar behavior to the blank FTO glass substrate, while the FeZr film gives an anodic current that is significantly greater than this “background” level (by a factor on the order of 2–5 times). Given that the data in Figure 6 is normalized to the geometric area, it was considered whether the differences are attributable to changes in the surface roughness and charges in the surface electrochemical double layer. While the electrochemically active surface area (ECSA) is not trivial to measure accurately, it was estimated in this work from the double-layer capacitance of these films (Figure S19). From the relative ECSAs evaluated using this method, the FeZr film has a roughness factor on the order of magnitude of 1.6 times that of the CuZr material. Surface roughness is therefore considered a significant component of these differences, but there are likely additional factors. The presence of redox processes before the catalytic wave in the cobalt and copper catalysts is consistent with the surface oxidation of the TM.^15,76,77^ These anodic waves are seen at ∼1.15 *V*_RHE_ for the CoZr film (Figure S17) and at ∼1.4 *V*_RHE_ for the CuZr film (Figure 6b). Interestingly, this feature was not observed in the FeZr film.

The three catalysts are active toward water oxidation. The FTO|CoZr film shows the best performance both in terms of onset potentials and Tafel slopes, while FTO|CuZr and FTO|FeZr show lower activities. The potential vs RHE of the FTO|CoZr film at current densities of 1 mA cm^–2^ is 0.3 V lower than that of the FTO|CuZr and FTO|FeZr films which are both very close (Figure 6a). The FTO|CoZr film exhibits OER overpotentials of ∼372 and ∼430 mV vs RHE at current densities of 1 and 10 mA cm^–2^, respectively. The FTO|FeZr and FTO|CuZr systems, however, fail to achieve high current densities and show higher overpotentials of ∼670 and 693 mV vs RHE at a current density of 1 mA cm^–2^. Furthermore, the Tafel slope of the FTO|CoZr film was 54 mV dec^–1^, 15–20 mV dec^–1^ lower than for the other two catalysts. The FTO|CoZr catalyst also shows an activity comparable to some of the best-performing zirconium-based systems reported that also show onset potentials below 1.6 V vs RHE and Tafel slopes of the order of 50 mV dec^–1^.^50,52^ Although it is worth noting that direct comparison of catalysts based on these metrics can be difficult due to differences in the measurement conditions,^17^ it is nevertheless an encouraging observation. More importantly, these findings confirm the higher intrinsic activity of cobalt compared with copper and iron as active sites for the water oxidation reaction. While this is in good agreement with previous studies that have shown cobalt oxides to have a higher intrinsic activity than other 3d TM oxides,^13,78^ this work represents a first examination of the role of 3d TMs as isolated catalytic sites incorporated in an inert host material.

The differences in current densities observed in the Tafel plot, particularly for the FTO|CoZr film, which shows a much higher current density at all potentials, can be attributed to differences in the surface area. In fact, the estimated ECSA differences from the double-layer capacitance measurements in Figure S19 suggest that the FTO|CoZr film has *a* relative roughness that is ∼15–25 times greater than the FTO|FeZr and FTO|CuZr films. The deviation from Tafel behavior at high potentials is attributed to diffusion limitation and local pH changes that can take place near the electrode under these conditions (Figure 6b). Finally, the stability of the catalysts during the operation was tested over a period of 18 h (Figure 6c). We found that the choice of 3d-TM dopant has an important effect on the stability of the catalyst. No significant degradation was found in the FTO|CoZr and in FTO|FeZr systems, which undergo an activation process showing an increase in current density of 78 and 76%, respectively, during the first 18 h of operation. On the other hand, a 7% decrease in current density for the FTO|CuZr system is seen, which is explained by the dissolution of copper, as supported by EDS (Figure 4), which shows a decrease in copper content after catalysis.

The amount of O_2_ produced during chronoamperometry was quantified for the FTO|CoZr system (Figure S18b). No initial CV catalyst activation was performed in this case, to aid the product quantification by avoiding additional O_2_ generation before the start of electrolysis. Three separate FTO|CoZr samples produced 12.6 ± 2.7 μmol cm^–2^ O_2_ over 4 h at 1.6 V vs RHE, with an average Faradaic yield (FY) of 57 ± 14% and a steady-state current density of 0.59 ± 0.27 mA cm^–2^ (recorded after 0.5 h under operation). The low FY suggested secondary electrochemical processes (such as oxidation of low valent Co species in the matrix, H_2_O_2_ production, etc.) contributing to the overall current density. The broader distribution in the values is due to morphological variations introduced during catalyst drop-casting.

To gain a better understanding of the role of the substrate and versatility of SSP deposition for the water oxidation reaction, the FTO|CoZr system was drop-casted onto three other substrates (graphite paper, carbon-fiber paper, and ITO-coated glass) and studied for electrochemical activity, as shown in Figure S20. The electrochemical performance for FTO and graphite paper was found to be similar, whereas the ITO-coated glass substrate showed lower activity. A lower onset potential and high current density were observed in the case of the carbon-fiber paper as the three-dimensional porous network provides a high surface area for catalyst deposition, resulting in a high ECSA value of 203.7 cm^2^ for CoZr on carbon-fiber paper (Figure S20c and Supporting Information).

In addition to their high electrocatalytic activity, SSP catalysts also allow more facile photoelectrode assembly compared to complex (photo)electrochemical deposition procedures. Accordingly, the CoZr film was evaluated as a suitable cocatalyst for solar-driven water oxidation by integrating it with the well-known light absorber BiVO_4_.^19,20^ For the PEC studies, the CoZr saturated solution was diluted using a 1:1 THF/EtOH mixture to different concentrations, 0.13, 0.052, 0.026, 0.013, and 0.0065 mol L^–1^, corresponding to 1:2, 1:5, 1:10, 1:20, and 1:40 dilutions, respectively, and spin-coated over BiVO_4_ films fabricated on FTO according to our previously reported procedure.^21,35^ Spin-coating yielded a fine and uniform layer of CoZr over BiVO_4_ without compromising its ability to absorb light (Figure S21). This FTO|BiVO_4_|CoZr photoanode was used as the working electrode.

**Figure 7:**
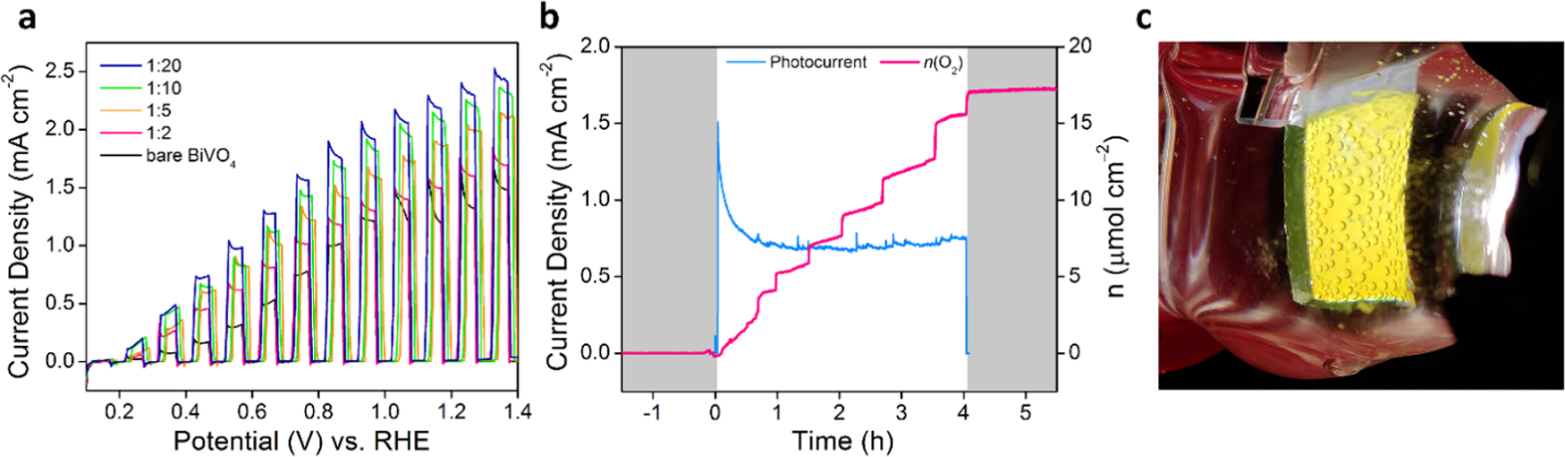
PEC responses of the FTO|BiVO_4_|CoZr system recorded in 0.1 M potassium borate (KBi), 0.1 M K_2_SO_4_ (pH 8.5) buffer solution at 25 °C without stirring. (a) Forward CV scans recorded under chopped simulated solar light irradiation (AM 1.5G, 100 mW cm^–2^, 1 sun) for the system with varying dilution ratios of the CoZr precursor solution. (b) Light-driven O_2_ evolution on a BiVO_4_|CoZr (1:20, 0.013 mol L^–1^) photoanode and corresponding photocurrent. The sample is maintained for 4 h at 1.23 V vs RHE in a 0.1 M KBi, 0.1 M K_2_SO_4_ (pH 8.5) buffer solution under continuous irradiation. Gray areas indicate no irradiation. (c) Photograph of a typical FTO|BiVO_4_|CoZr working electrode in operation under illumination.

BiVO_4_ is a photoanode with suitable onset potential for tandem water-splitting applications that operate under pH-neutral conditions. A 0.1 M KBi buffer (pH 8.5) with 0.1 M K_2_SO_4_ as a supporting electrolyte was used for the PEC measurements. The PEC performance of the different samples produced by spin-coating using different concentrations of **1** is shown in Figure 7. It was observed that the PEC performance increases with the dilution of the solution of **1**, with photocurrent densities (at 1.23 V vs RHE) increasing from 1.64 mA cm^–2^ for a 1:2 (0.13 mol L^–1^) sample to 2.41 mA cm^–2^ for a 1:20 (0.013 mol L^–1^) sample. The FTO|BiVO_4_|CoZr (1:20) film showed the highest PEC activity with an early onset potential of 0.21 V vs RHE, while bare BiVO_4_ showed a maximum photocurrent density of only 1.62 mA cm^–2^ at 1.23 V vs RHE and a higher onset potential of 0.33 V vs RHE. The presence of active Co sites over the BiVO_4_ surface helps to reduce the onset potential for water oxidation and facilitates the extraction of holes from the valence band of the BiVO_4_ generated upon illumination. This improves the photocurrent density by significantly reducing the charge recombination processes.^24,26^ However, spin-coating of more concentrated solutions produces thicker CoZr layers, which results in poor light absorption by the BiVO_4_ layer and reduces the efficiency of charge transport to the electrolyte interface. Moreover, increasing the dilution to 1:40 reduces the photocurrent density owing to the decrease in the Co active sites (Figure S22f).

The PEC O_2_ detection was conducted for the best-performing 1:20 CoZr diluted sample (Figure 7b). A triplicate of the BiVO_4_|CoZr (1:20) samples sustained a steady-state photocurrent of 0.71 ± 0.06 mA cm^–2^, producing 17.1 ± 0.3 μmol cm^–2^ O_2_ with an FY of 72 ± 6% over the course of 4 h at 1.23 V vs RHE, with the smaller variation in performance and improved Faradaic yield owing to the more homogeneous catalyst deposition. The stepwise increase in the detected oxygen amount could be traced back to O_2_ bubble formation and release from the photoanode surface, which supports the influence of gas trapping on the observed Faradaic yield.

## 4 Conclusions

We have synthesized Zr-based cages containing copper, iron, and cobalt as “dopants” and observed that their structures are strongly dependent on the coordination characteristics of the dopant atoms. We demonstrate that these precursor molecules are versatile reagents for low-cost fabrication of catalytically active doped-zirconia films over a wide variety of substrates. EDS and XPS analysis show that these films are indeed zirconia doped with first-row TMs. All of them show activity toward water oxidation with the FTO|CoZr system having lower Tafel slopes and onset potentials compared to FTO|CuZr and FTO|FeZr. This demonstrates the essential role of the dopant in the catalytic activity of these materials and confirms the higher intrinsic activity of cobalt compared to iron and copper ions as active sites in an inert zirconia matrix. Finally, the integration of the CoZr film with BiVO_4_ by spin-coating led to a significant enhancement in the photoelectrocatalytic performance of this material, lowering the onset potentials by 0.1 V and increasing the photocurrent densities by 1.2 mA cm^–2^, proving that single-source precursors represent a simple yet effective approach for the deposition of catalytically active films that can act on their own or as cocatalysts enhancing the activity of known materials. This method represents a step forward in the development of large-scale multijunction devices that could incorporate electrocatalysts, light absorbers, and spectral converters that will allow efficient utilization of sunlight for chemical energy storage.

## Supporting Information Available

Details of the single-crystal structures and refinements, SEM images and EDS analysis of the catalytic films, FT-IR spectra of the precursor molecules, XPS measurements and details on the calibration procedure, (photo)electrochemical data including EIS and ECSA measurements, and ICP-OES results (PDF)

## Accession Codes

CCDC 2011723–2011725 contain the supplementary crystallographic data for this paper. These data can be obtained free of charge via www.ccdc.cam.ac.uk/data_request/cif, or by emailing data_request@ccdc.cam.ac.uk, or by contacting The Cambridge Crystallographic Data Centre, 12 Union Road, Cambridge CB2 1EZ, UK; fax: +44 1223 336033.
