# Supplementary material for: Single-Source Deposition of Mixed-Metal Oxide Films Containing Zirconium and 3d Transition Metals for (Photo)electrocatalytic Water Oxidation
Source: Inorg Chem. 2022 Apr 12;61(16):6223–33. doi: 10.1021/acs.inorgchem.2c00403 (PMC9098167; doi:10.1021/acs.inorgchem.2c00403)
Supplement: Supplementary file 1 — ic2c00403_si_001.pdf [file ic2c00403_si_001.pdf]

Supporting Information

Single-source Deposition of Mixed-metal Oxide Films Containing Zirconium and 3d Transition Metals for (Photo)electrocatalytic Water Oxidation

Victor Riesgo-Gonzalez,[†, ⁂, ‡] *Subhajit Bhattacharjee,*[†, ‡] *Xinsheng Dong,*[§, ‡] *David S. Hall,*[†, ⁂] *Virgil Andrei,*[†] *Andrew D. Bond,*[†] *Clare P. Grey,*[†, ⁂] *Erwin Reisner,*[†, *] *Dominic S. Wright.*[†, ⁂, *]

[†] Yusuf Hamied Department of Chemistry, University of Cambridge, Lensfield Rd, Cambridge CB2 1EW, United Kingdom

[§] College of Chemical Engineering, Nanjing Forestry University, Nanjing, Jiangsu 210037, China

[⁂] The Faraday Institution, Quad One, Harwell Science and Innovation Campus, Didcot OX11 0RA, United Kingdom

1. Crystallographic data

1.1. Crystal Structure and Refinement of 1

The crystal structure of $[\{Zr_4(\mu_4\text{-}O)(OEt)_{15}\}Co^{II}Cl]$ (denoted **1**) is isomorphous with the published Zn compound, $[\{Zr_4(\mu_4\text{-}O)(OEt)_{15}\}Zn^{II}Cl]$[S1].The refinement of the Zn compound (denoted **1**_Zn) was noted by the authors to be problematic, and the refinement in the present work of **1** was equally problematic.

The structure of **1**_Zn was described in space group $P2_1/n$ (without disorder), but the note in the published CIF indicates streaking of the diffraction pattern and probable twinning. The *R*-factor for refinement of **1**_Zn was *ca* 15%. The diffraction pattern of **1** showed similar features (Figure S1). The best description in this case was produced in space group *C*2/*c*. The complex is situated on a crystallographic 2-fold rotation axis (passing through atoms O1 and O5), which necessitates 50% site occupancy for atoms Co1 and Cl1. Refinement of the site occupancies confirms these values. Lowering the symmetry to *Cc* or $P2_1/n$ does not resolve this disorder, so the *C*2/*c* description is retained. The ethoxide groups are in general poorly resolved and the displacement parameters of all C atoms are large. Geometrical and ISOR restraints were applied. Alternative trials with the displacement parameters linked to common isotropic values led to quite significant increases in the *R*-factors, so the restrained anisotropic model was finally preferred.

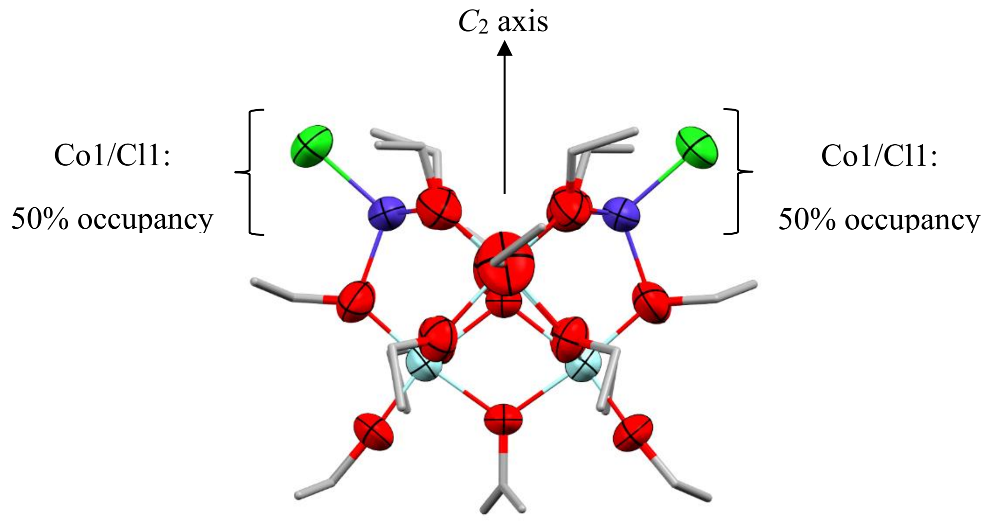

Figure S1. Crystal structure of **1**.

Data for the previously published **1**_Zn were collected in our laboratory (in 2010), so processed data (not CCD frames) were available for re-examination. [S1] The data suggest that the ZnCl structure appeared more clearly to be primitive (with the same unit-cell parameters ≈ 22.2, 11.2, 22.9, 90, 118.3, 90), but streaking/twinning was noted for the diffraction pattern, and the refinement was similarly quite poor. The conclusion from **1** and **1**_Zn is that this structure type is prone to twinning/stacking disorder, and the *C*2/*c* structure presented for **1** is a representation of the average structure. The chemical identity of **1** and **1**_Zn, and in particular the presence of only one (Co/Zn)Cl group per complex, is clear.

1.2. Crystal Structure and Refinement of 2

Refinement of $[\{Zr_4(\mu_4\text{-}O)_2(EtO)_{16}\}(Fe^{III}Cl)_2]\cdot 2THF$, denoted **2**·2THF, was largely straightforward. Geometrical and ISOR restraints were applied to all ethoxide groups, some of which are modelled as disordered. The THF molecule (one in the asymmetric unit, two per

centrosymmetric complex **2**) is quite poorly resolved. Assignment of the O atom within the 5-membered ring was based on initial indications from the displacement parameters, but it is highly uncertain. The geometry of the ring is restrained, and isotropic displacement parameters are constrained to a single common value in the final refinement. Eliminating this problematic THF molecule and applying *SQUEEZE* led to some improvement in the *R*-factors ($R1 = 0.032$, $wR2 = 0.083$), but inclusion of THF was finally preferred for chemical clarity.

1.3. Crystal Structure and Refinement of 3

Data collection for **3** was carried out on several crystals taken from different batches, in all cases giving comparable results. The crystals appear to be twinned, and the final refinement is based on a 2-component (HKLF-5) refinement, resolved post-integration using *PLATON/TWINROTMAT* (A. Spek, Utrecht University). The refinement produced good *R*-factors and a well-defined centrosymmetric complex with formula $[\{Zr_4(\mu_4\text{-}O)_2(EtO)_{16}\}\{(Cu^{II}Cl)_2(OEt)\}_2]$ (Figure S2).

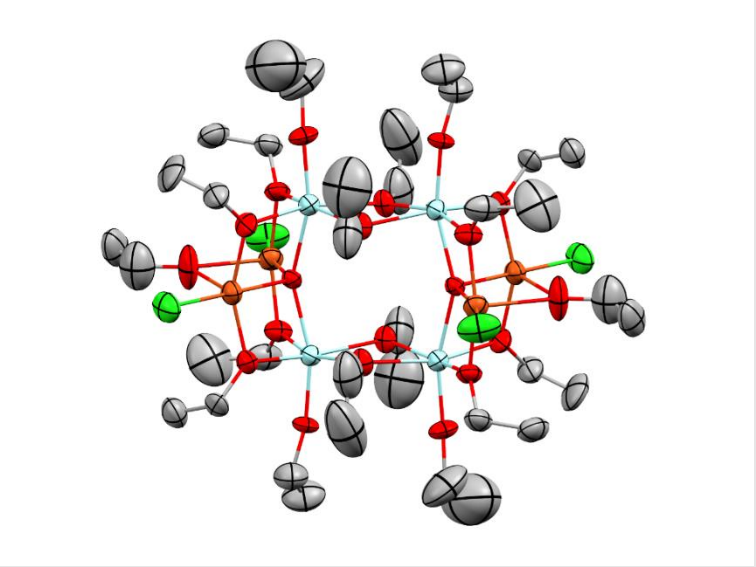

Figure S2. Crystal structure of **3**.

This appears to be a dianion, but with no clear charge-balancing cation(s). The structure contains additional electron density in spaces between the complexes, which is modelled as one disordered ethanol molecule per void. Omitting this molecule and applying *SQUEEZE* corrects for 130 electrons per unit cell, which gives *ca* 32 electrons per void space, broadly consistent with the 26 electrons expected for EtOH. Inclusion of any isolated charge-balancing cation in this site (*e.g.,* Na^+ or H_3O^+) is unlikely on the basis of the clearly hydrophobic environment of the channels.

Several possibilities exist for charge balancing within the crystal structure:

1. Two H^+ associated with the complex: there is no clear location to accommodate these protons. In the *SQUEEZE* result (which provides the highest precision for the main complex), the two non-bridging EtO^- ligands have Zr–O = 1.907 (6) and 1.917 (6) Å, compared to protonated examples in the CSD with mean Zr–O ≈ 2.25 Å. Likewise, the geometry of the bridging EtO^- ligands and the central [O] atoms do not indicate any protonation sites.

2. Exchange of some Cu–Cl groups for Cu–H_2O: although the solvents used were nominally dried, it is possible that H_2O could be present. Cu–Cl/H_2O exchange is quite commonly seen in crystal structures, with the Cl and O atoms occupying essentially the same atomic site. However, the X-ray data do not support this hypothesis. Producing mixed Cl/O sites and refining site occupancy factors invariably produced fully occupied Cl atoms. The displacement ellipsoids of Cl show no indication of partial occupancy.

3. Exchange of some EtO^- ligands for H_2O: this seems the most probable mechanism for charge balance, given the X-ray data. In the X-ray refinement, the coordinated O atoms would be largely unaffected (although Zr–OH_2 bond distances would be longer than Zr–OEt), but the displacement parameters of the C atoms of EtO^- should indicate partial site occupancy. This is very difficult to assess, since the EtO^- ligands are anyway poorly resolved, and the displacement parameters of the C atoms are all large. Some are clearly larger than others, but it is difficult to distinguish potential fractional occupancy from positional disorder. Short contacts do exist between some EtO^- ligands in neighboring complexes (most notably atoms C82 and C102). Bridging Zr–H_2O–Zr units are rare in the CSD (only one example), while non-bridging Zr–OH_2 is common. It is possible that some terminal EtO^- ligands are replaced by water in a manner that does not produce crystallographic order. Replacement of the ethoxide ligand in Cu–OEt–Cu might also be possible; this is difficult to assess from the geometry (since 5-coordinate Cu^{II} is highly variable), and the displacement ellipsoids are not clearly larger than any of the other EtO^- ligands.

Crystallographic data and refinement results are summarized in **Table S1**.

Table S1. Crystallographic data and refinement of complexes **1**, **2** and **3**.

	1	2	3
CCDC number	2011723	2011724	2011725
Cambridge data number	DW_K1_0004	DW_K1_0007	DW_K1_0006
Chemical formula	$C_{30}H_{75}ClCoO_{16}Zr_4$	$C_{40}H_{96}Cl_2Fe_2O_{20}Zr_4$	$C_{38}H_{96}Cl_4Cu_4O_{21}Zr_4$
Formula weight	1151.16	1444.64	1649.98
Temperature / K	180(2)	180(2)	180(2)
Crystal system	monoclinic	triclinic	monoclinic
Space group	*C*2/*c*	*P*–1	*C*2/*c*
a / Å	22.2252(6)	10.8605(4)	27.6397(7)
b / Å	11.1515(4)	11.8055(4)	13.3575(4)
c / Å	22.8685(8)	13.4100(5)	18.1081(6)
α / deg	90	95.6906(13)	90
β / deg	118.2860(13)	92.7536(13)	101.0883(12)
γ / deg	90	112.3541(15)	90
Unit-cell volume / $Å^3$	4991.0(3)	1575.42(10)	6560.7(3)
Z	4	1	4
Calc. density / g cm^{-3}	1.532	1.523	1.670
F(000)	2348	742	3344
Radiation type	MoKα	MoKα	MoKα
Absorption coefficient / mm^{-1}	1.241	1.231	2.103
Crystal size / mm^3	0.35 x 0.23 x 0.12	0.32 x 0.28 x 0.06	0.28 x 0.18 x 0.14
2θ range / deg	7.05-43.93	7.03-50.00	7.36-43.97
Completeness to max 2θ	0.986	0.982	0.991
No. of reflections measured	11821	13683	14557

No. of independent reflections	3000	5421	14557
R(int)	0.0403	0.0461	0.0636
No. parameters / restraints	250 / 120	316 / 160	335 / 159
Final $R1$ values ($I > 2\sigma(I)$)	0.0868	0.0409	0.0526
Final $wR(F^2)$ values (all data)	0.1001	0.0568	0.0774
Goodness-of-fit on F^2	1.079	1.029	0.977
Largest difference peak & hole / e $Å^{-3}$	1.097, -0.605	0.724, -0.506	0.931, -0.477

Table S2. Selected bond-lengths

Precursor	Bond	Bond length / Å
CoZr	Zr•••Zr	3.459(2)–3.525(2)
	Terminal Zr-O(Et), Zr-bridging Zr-O(Et)	1.866(10)-2.202(8)
	Zr-O_{oxo}	2.213(3)-2.234(5)
	Co-O(Et)	1.965(10)-2.196(12)
	Co-Cl	2.242(7)
	Zr•••Zr•••Zr	59.36(3)-61.29(4)
FeZr	Zr•••Zr	3.537(1)
	Terminal Zr-O(Et), Zr-bridging Zr-O(Et)	2.149(3)-2.287(3)
	Zr-O_{oxo}	2.113(3)-2.173(3)
	Fe-O_{oxo}	1.992(3)
	Fe-O(Et)	1.915(3)-1.967(3)
	Fe-Cl	2.246(1)
	Zr•••Zr•••Zr	62.11(1)-117.89(1)
CuZr	Zr•••Zr [Zr(μ_3-O)Zr]	4.172(1)
	Zr•••Zr [Zr(μ_2-OEt)$_2$]	3.496(2)
	Zr-O(Et)	1.905(6)-1.907(5)
	Zr-bridging Zr-μ-O(Et)	2.145(6)-2.158(7)
	Zr-O_{oxo}	2.136(5)-2.152(5)
	Cu-O_{oxo}	2.051(6)-2.066(6)
	Cu-O(Et)(bridging Zr)	1.943(6)-1.948(6)
	Cu-OEt (bridging Cu)	2.439(8)-2.490(8)
	Cu-Cl	2.202(3)-2.208(3)
	Zr•••Zr•••Zr	88.83(2)-91.17(2)

2. Chemical Analysis of the Catalyst Films

2.1. EDS elemental analysis of the fresh and cycled electrodes:

To analyze the elemental composition and distribution of elements in the catalysts, EDS point scans were taken across the sample. The normalized atomic percentage of each of the elements detected by EDS is presented and the dopant : Zr ratio is calculated. Outliers were removed using a Dixon's Q test with 80 % confidence. The 95 % confidence intervals were calculated.

2.2.1. EDS analysis of CoZr electrode before catalysis:

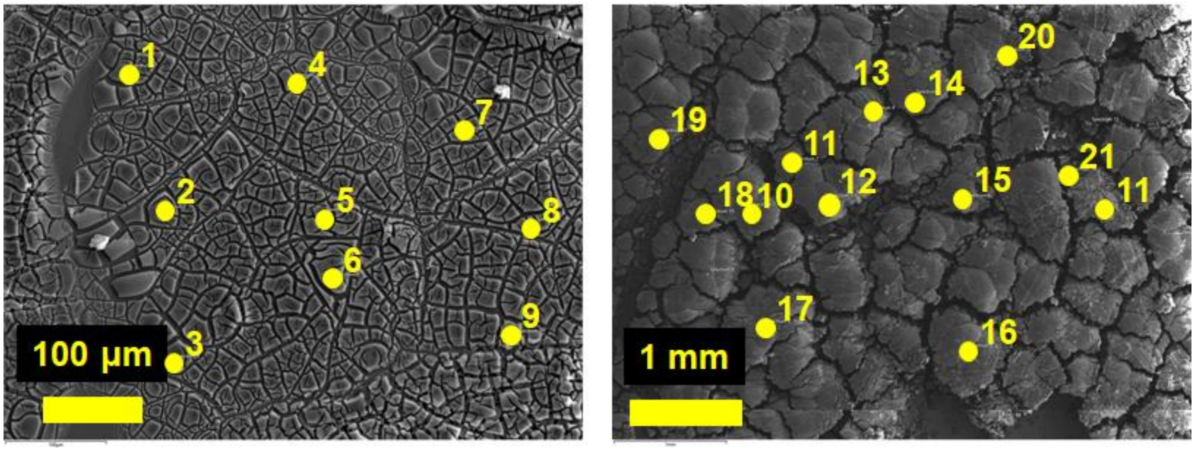

Figure S3. Location of the point scans measured on the CoZr electrode before catalysis.

Table S3. Normalized atomic percentages of the elements detected in the CoZr electrode before catalysis.

	Atomic %					
Spectrum	O	C	Zr	Cl	Co	Co : Zr
1	63.7	17.2	11.9	5.4	1.8	0.15
2	63.5	15.3	13.2	5.9	2.0	0.15
3	63.6	15.0	13.4	6.1	1.9	0.14
4	62.8	21.8	9.7	4.2	1.5	0.15
5	59.5	15.8	15.5	6.7	2.5	0.16
6	58.4	16.7	15.8	6.7	2.4	0.15
7	59.2	23.6	10.8	4.7	1.6	0.15
8	59.7	19.2	13.5	5.5	2.1	0.16
9	58.1	21.7	12.7	5.7	1.9	0.15
10	69.1	21.4	6.9	2.3	0.2	0.03
11	61.1	17.5	14.0	4.8	2.5	0.18
12	75.6	15.7	6.6	2.1	0.1	0.02
13	66.9	19.1	9.7	3.7	0.6	0.06
14	62.7	16.1	14.2	5.7	1.3	0.090
15	70.1	17.4	8.8	3.2	0.4	0.05
16	65.9	17.4	11.1	4.3	1.2	0.11
17	59.8	16.8	15.5	6.0	1.8	0.12
18	61.9	18.0	13.5	4.7	1.9	0.14
19	62.8	15.0	14.5	6.0	1.6	0.11
20	60.6	19.8	12.6	4.7	2.3	0.18
21	58.6	22.8	12.5	4.4	1.7	0.14
Average	63.0	18.3	12.2	4.9	1.6	0.10
Confidence interval	1.67	1.00	1.0	0.49	0.27	0.02

2.2.2. EDS analysis of CoZr electrode after catalysis:

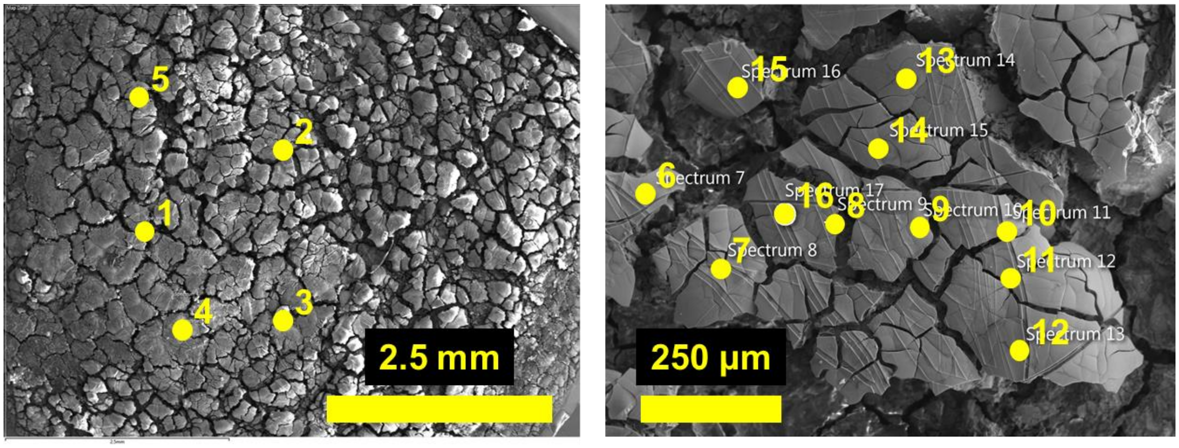

Figure S4. Location where the point scans were measured on the CoZr electrode after catalysis.

Table S4. Normalized atomic percentages of the elements detected in the CoZr electrode after catalysis.

	Atomic %						
Spectrum	O	C	Zr	K	F	Co	Co :Zr
1	48	19.5	18.8	4.9	5.5	3.4	0.18
2	33.7	0	11.1	5.2	47.1	2.7	0.24
3	34.4	0	12.4	6.1	41.9	4.5	0.36
4	25.1	0	10.5	4.1	58.4	1.9	0.18
5	40.1	31.5	8.7	3.5	15	1.2	0.14
6	69.8	11.3	14.9	1.8	0	2.2	0.15
7	64.4	17	10.4	3.4	0	4.8	0.46
8	53.5	28	14.1	2.1	0	2.4	0.17
9	68.1	10	17.2	2.1	0	2.6	0.15
10	63.6	16.5	15.4	2.2	0	2.3	0.15
11	53.9	26.4	13.5	2.8	0	3.4	0.25
12	46.8	31.3	16.5	2.5	0	2.8	0.17
13	62.2	10.3	21.2	2.9	0	3.3	0.16
14	64.5	12.3	18.2	2.3	0	2.7	0.15
15	56.7	11.3	24.4	3.1	0	4.5	0.18
16	62.5	16.9	15.9	2.2	0	2.6	0.16
Average	53	15.1	15.2	3.2	10.5	3	0.2
Confidence interval	5.88	4.47	1.81	0.55	19.8	0.43	0.04

2.2.3. EDS analysis of CuZr electrode before catalysis

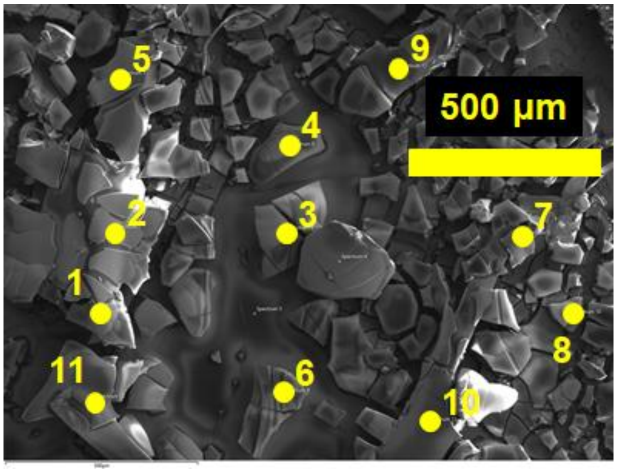

Figure S5. Location where the point scans were measured on the CuZr electrode before catalysis.

Table S5. Normalized atomic percentages of the elements detected in the CuZr electrode before catalysis.

	Atomic %					
Spectrum	O	C	Zr	Cl	Cu	Cu : Zr
1	64.9	25.6	6.5	1.2	1.8	0.28
2	69.6	15.6	10.6	2.1	2.2	0.21
3	23.8	65.1	8.7	1.7	0.6	0.07
4	49.4	37.4	9.6	1.7	1.9	0.2
5	70.0	17.6	8.6	1.6	2.2	0.26
6	47.8	42.5	7.1	1.1	1.5	0.21
7	47.3	46.4	4.1	0.6	1.7	0.41
8	60.6	23.5	11.6	2.2	2.1	0.18
9	33.3	57.7	6.9	1.0	1.1	0.16
10	73.2	15.2	8.5	1.8	1.2	0.14
11	69.3	19	8.3	1.6	1.9	0.23
Average	55.4	33.2	8.2	1.5	1.7	0.2
Confidence interval	8.88	9.58	1.1	0.26	0.28	0.04

2.2.4. EDS analysis of CuZr electrode after catalysis

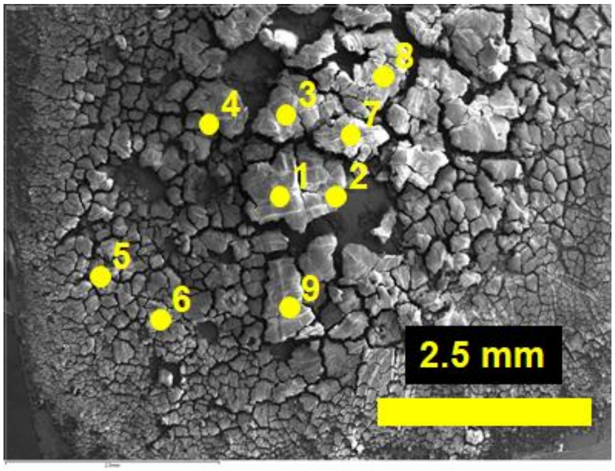

Figure S6. Location where the point scans were measured on the CuZr electrode after catalysis.

Table S6. Normalized atomic percentages of the elements detected in the CuZr electrode after catalysis.

	Atomic %					
Spectrum	O	C	Zr	K	Cu	Cu : Zr
1	57.9	30.1	6.6	4.8	0.5	0.08
2	57.9	20.6	14.1	6.1	1.2	0.085
3	57.4	24.4	11.8	5.3	1.2	0.10
4	59.7	28.7	7.6	2.7	1.3	0.17
5	54.7	25.8	13.2	5.0	1.3	0.10
6	75.1	0	17.6	5.3	2.0	0.11
7	58.6	27.7	12.4	0	1.3	0.10
8	54.3	24.3	14.5	5.8	1.1	0.076
9	59.0	15.9	17.9	5.9	1.4	0.078
Average	59.4	21.9	13	4.5	1.0	0.10
Confidence interval	3.87	5.84	2.4	1.3	0.3	0.04

2.2.5. EDS analysis of FeZr electrode before catalysis

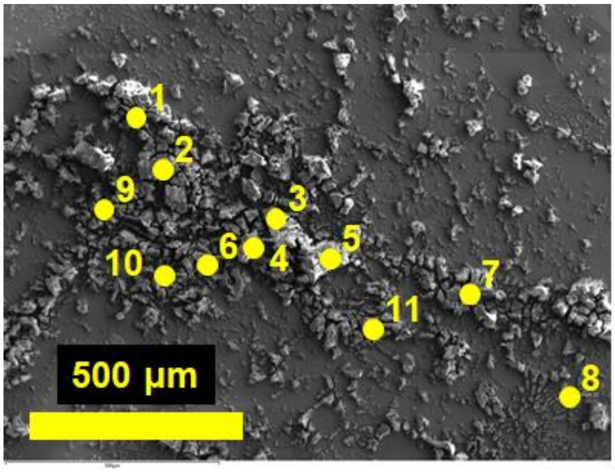

Figure S7. Location where the point scans were measured on the FeZr electrode before catalysis.

Table S7. Normalized atomic percentages of the elements detected in the FeZr electrode before catalysis.

	Atomic %				
Spectrum	O	Cl	Zr	Fe	Fe : Zr
1	63.7	7.2	25.1	4.0	0.16
2	74.4	6.0	17.1	2.5	0.15
3	78.1	5.7	14.8	1.4	0.095
4	66.1	6.7	23.0	4.2	0.18
5	64.7	7.5	24.4	3.4	0.14
6	67.2	7.3	22.5	2.9	0.13
7	79.6	5.6	13.5	1.3	0.096
8	77.3	5.9	15.3	1.5	0.098
9	73.8	6.2	17.9	2.1	0.12
10	69.2	7.3	20.8	2.7	0.13
11	69.5	7.0	21.4	2.1	0.098
Average	71.2	6.6	19.6	2.6	0.13
Confidence intervals	3.06	0.39	2.2	0.54	0.031

2.2.6. EDS analysis of FeZr electrode after catalysis

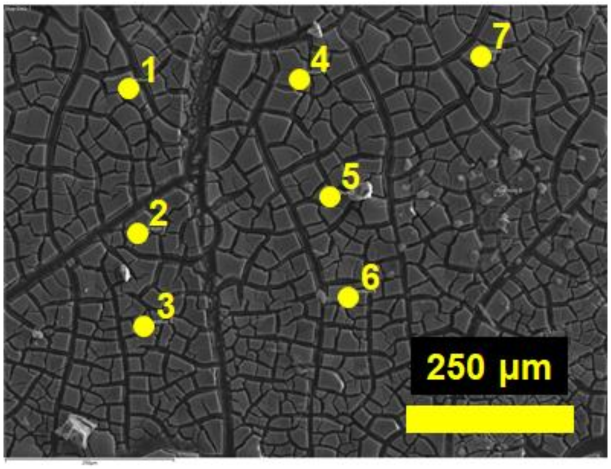

Figure S8. Location where the point scans were measured on the FeZr electrode after catalysis.

Table S8: Normalized atomic percentages of the elements detected in the FeZr electrode after catalysis.

	Atomic %					
Spectrum	O	Zr	C	K	Fe	Fe : Zr
1	71.8	21.0	0	4.9	2.4	0.11
2	60.6	16.3	17.5	3.8	1.8	0.11
3	60.2	15.6	18.8	3.7	1.7	0.11
4	61.5	15.5	17.9	3.6	1.5	0.10
5	56.9	16.2	21.3	3.7	1.9	0.12
6	59.6	14.8	20.3	3.6	1.6	0.11
7	62.7	16.5	15.3	3.8	1.7	0.10
Average	61.9	16.6	15.9	3.9	1.8	0.11
Confidence intervals	3.38	1.46	5.20	0.33	0.21	0.016

2.2.7. Summary of the EDS results and dopant : Zr ratio

Table S9. Summary of EDS – derived dopant : Zr ratios

Sample	TM : Zr ratio	Confidence interval
Co : Zr precursor	0.25	-
Co : Zr before	0.12	0.02
Co : Zr after	0.2	0.04
Cu : Zr precursor	1	-
Cu : Zr before	0.2	0.04
Cu : Zr after	0.1	0.04
Fe : Zr precursor	0.5	-
Fe : Zr before	0.13	0.031
Fe : Zr after	0.11	0.016

2.3. Infrared spectroscopy

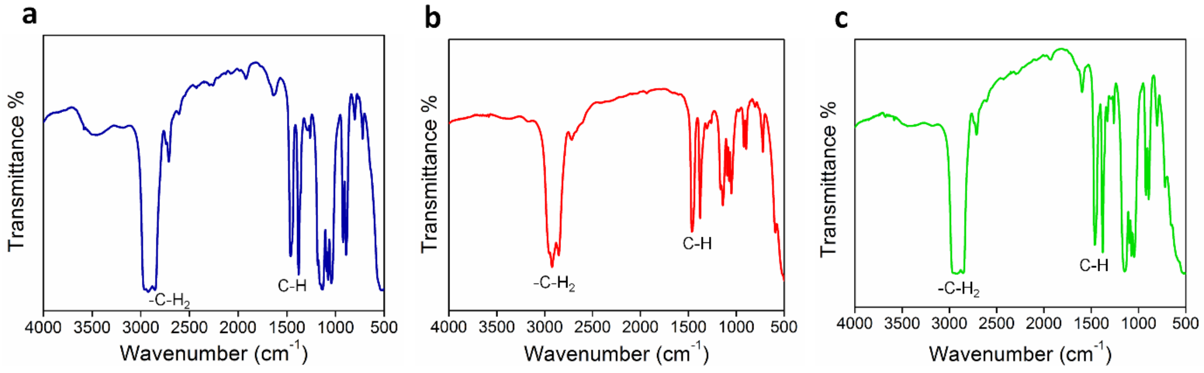

Figure S9. FT-IR spectra of precursors (a) **1**, (b) **2**, and (c) **3**.

2.4. X-ray Photoelectron Spectroscopy

2.4.1. X-ray Photoelectron Spectroscopy (XPS) Calibration and Fitting

Measured XPS binding energies were adjusted to compensate for the shift that arises from accumulated electrostatic charge by setting the alkyl hydrocarbon C 1s peak (from adventitious carbon) to the binding energy of 284.8 eV. This was performed fitting four peaks corresponding to alkyl (C–H, C–C), ethers and alcohols (C–O–C,C–OH), carbonyl (C=O), and esters and carbonates (O–C=O); the peaks were constrained to have equal width and their spacing was 1.5 eV, 3.0 eV and 4 – 5 eV, relative to the alkyl peak, respectively.[S2] The C 1s region in the samples prepared in Nafion posed challenges due to the large number of carbon chemical environments, as well as overlap with the K 2p peak from potassium inclusions in the Nafion from exposure to KOH solution. A C–F peak was added – its width was allowed to vary freely, and its position was set at ~8 eV above the alkyl peak. The K 2p was fitted as a single peak with freely varied width and a position set at ~11 eV above the alkyl peak. Example fitted spectra are shown for the free-standing materials in **Figure S10** and for materials that have been prepared with Nafion and immersed in aqueous KOH solution in **Figure S11**.

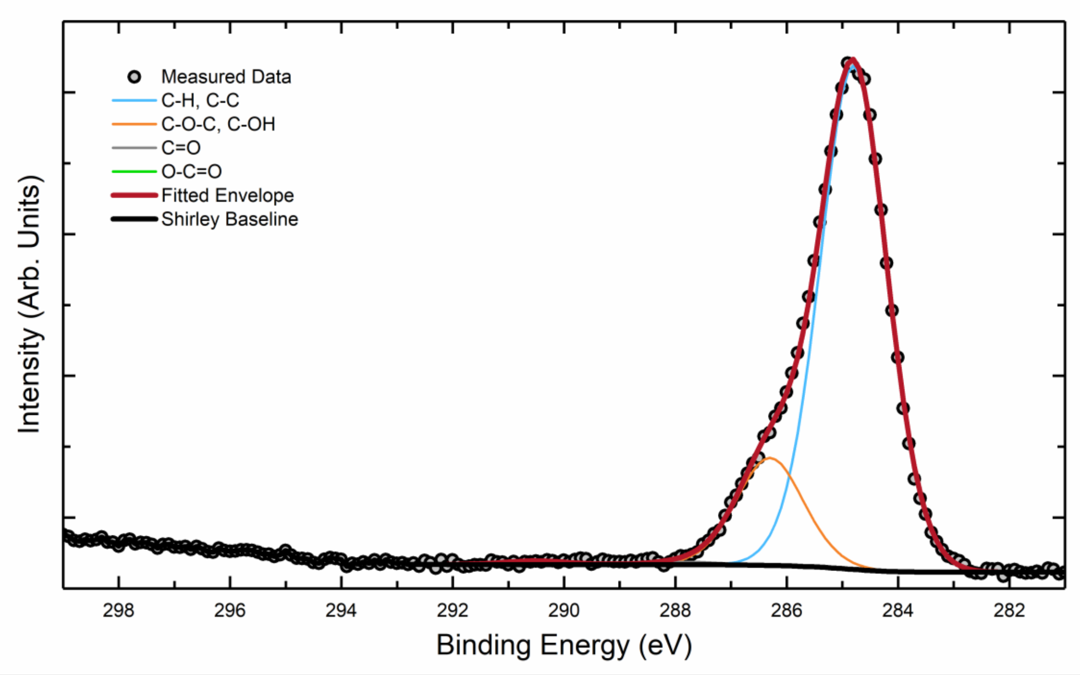

Figure S10. Fitted C 1s region of the CoZr sample, demonstrating the peaks used for charge compensation of free-standing catalyst materials in this work.

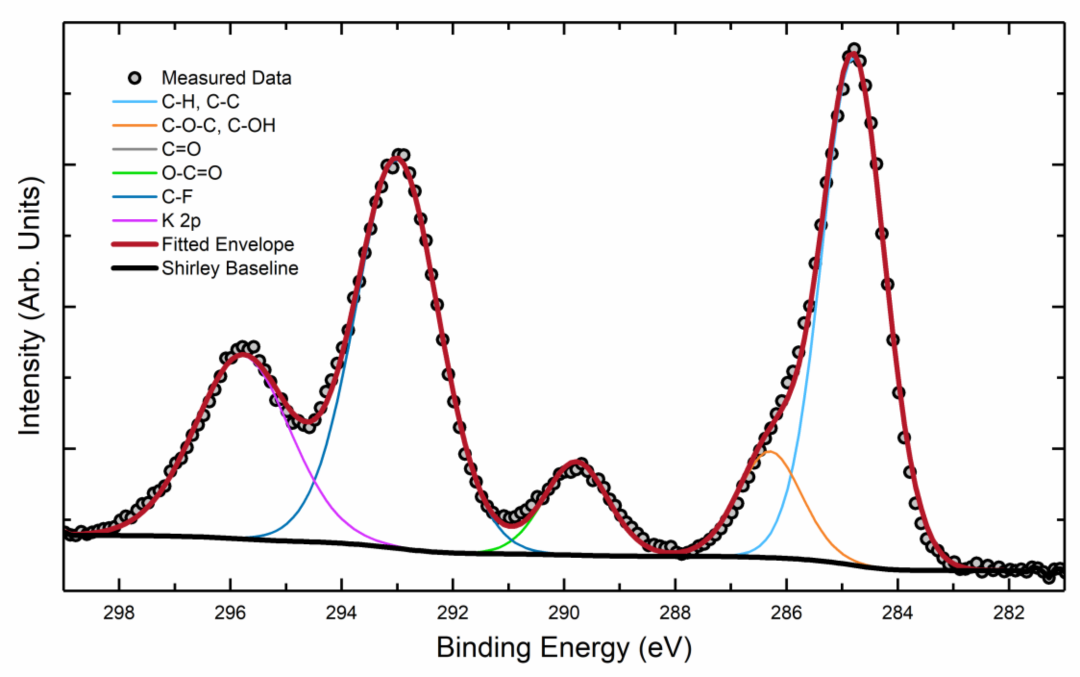

Figure S11. Fitted C 1s region of the FeZr/Nafion sample, demonstrating the peaks used for charge compensation of catalyst materials prepared with Nafion and immersed in aqueous KOH.

2.4.2. X-ray Photoelectron Spectroscopy (XPS) Survey and High-Resolution Spectra

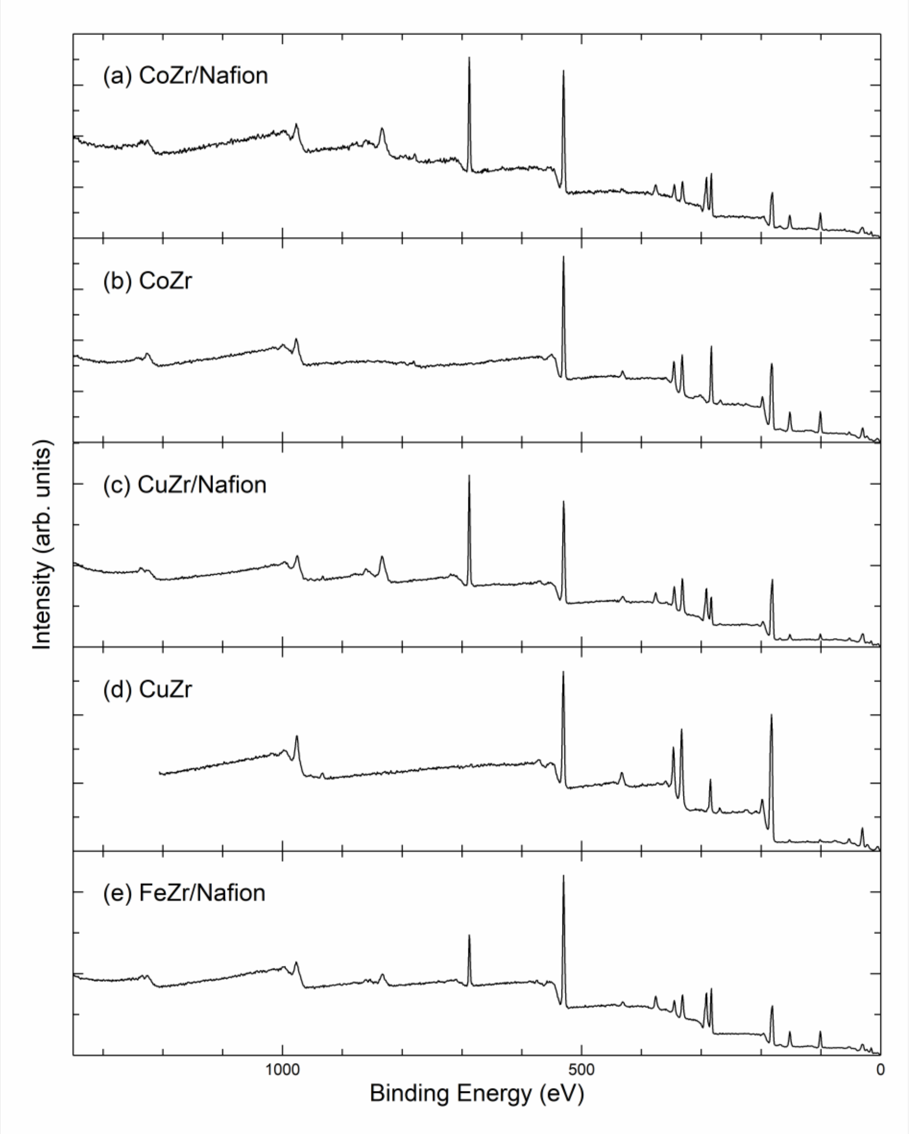

Figure S12. XPS survey spectra for the materials characterized in this work. Note that (d) was measured on a different instrument. During the fabrication of the electrocatalyst film, a 1:1 Nafion : EtOH mixture was drop-casted over the catalytic films to improve their adherence to the FTO glass. For the XPS measurements, samples with and without Nafion were used. The ZR 3d spectra shown in the main text correspond to the samples treated with a 1:1 solution of Nafion in ethanol (see experimental section).

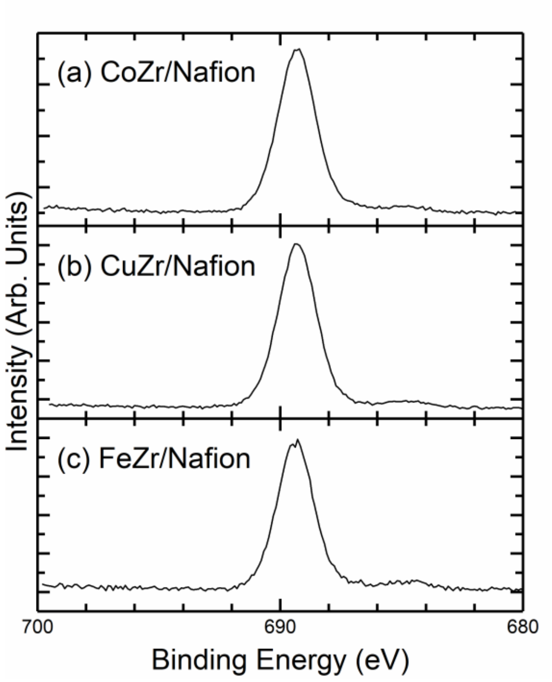

Figure S13. High-resolution XPS F1s region for the catalyst materials prepared with Nafion and immersed in aqueous KOH.

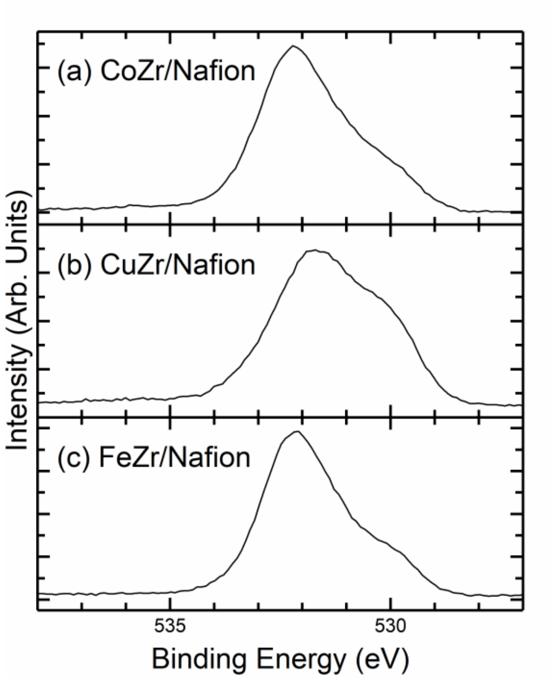

Figure S14. High-resolution XPS O1s region for the catalyst materials prepared with Nafion and immersed in aqueous KOH.

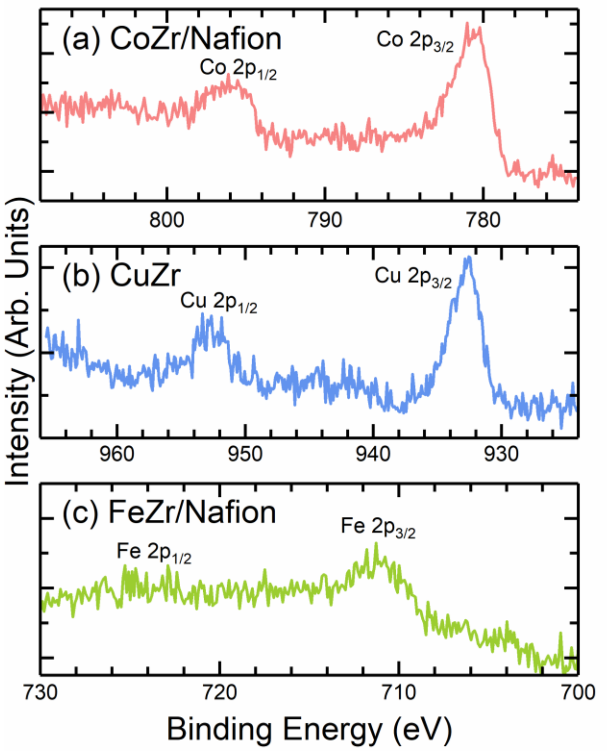

Figure S15. High-resolution XPS spectra of the Co 2p , Cu 2p and Fe 2p regions for a) FTO|CoZr, b) FTO|CuZr, and c) FTO|FeZr samples. Spectra a) and c) were taken from the Nafion-treated samples and spectrum b) was obtained from the as-deposited CuZr film i.e., before Nafion addition.

Table S10. XPS fit parameters for the Zr 3d region, measured at a pass energy of 20 eV.

Sample	Zr $3d_{5/2}$ Peak 1 [eV]	FWHM [eV]	$3d_{5/2}$ – $3d_{3/2}$ Splitting [eV]	Zr $3d_{5/2}$ Peak 2 [eV]	FWHM [eV]	$3d_{5/2}$ – $3d_{3/2}$ Splitting [eV]	Peak 2 Area [%]
CoZr/Nafion	182.26	1.36	2.37	183.81	2.24	2.27	27.9
CuZr/Nafion	182.19	1.40	2.39	183.15	1.92	2.31	22.7
FeZr/Nafion	182.22	1.31	2.37	183.59	1.76	2.38	31.6

3. Electrochemical Characterization

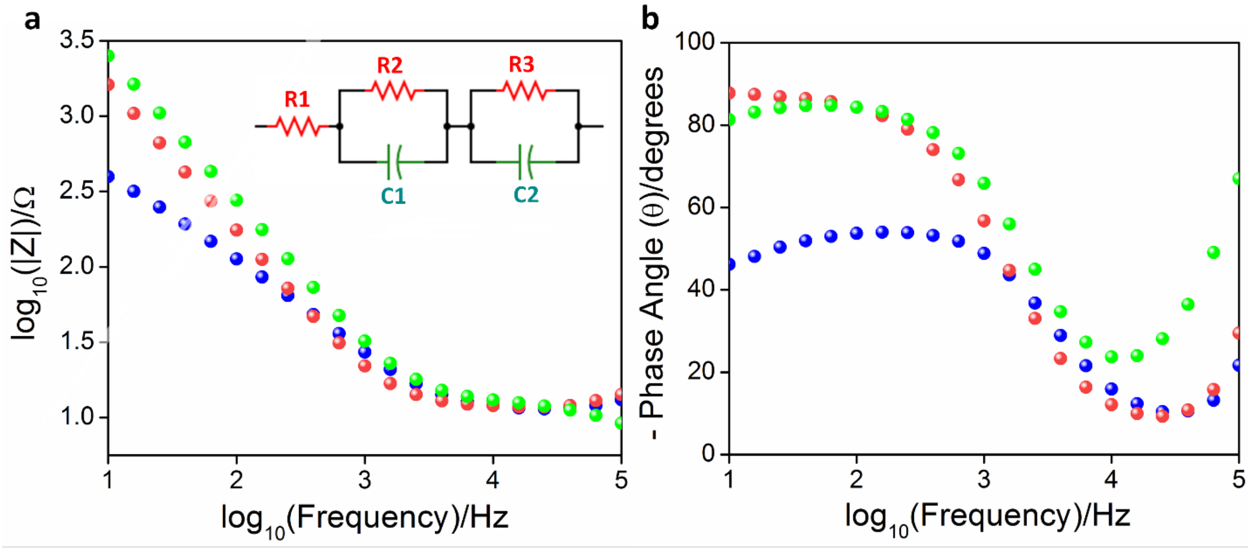

Figure S16. Electrochemical impedance spectroscopy data for CoZr (blue), FeZr (red) and CuZr (green) systems. (a) Bode plots showing the variation of log Z vs. log (frequency) with the (inset) equivalent circuit (R indicates resistance, C indicates capacitance). (b) Variation of the phase angle with log (frequency) for the respective systems.

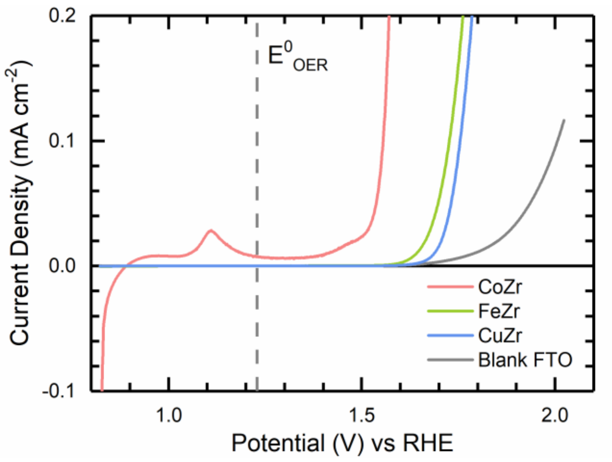

Figure S17. Linear sweep voltammetry (LSV) was measured on the untreated FTO glass (grey) and on the FTO|CoZr (red), FTO|FeZr (green), and FTO|CuZr (blue) coatings (scan rate 5 mV s^{-1}). The CoZr film presents an anodic wave at ~1.15 V_{RHE}, below the OER standard potential.

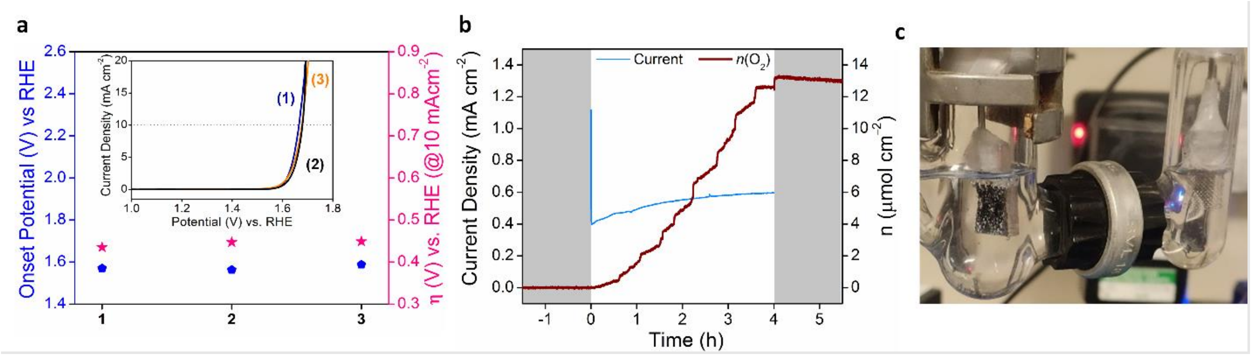

Figure S18. (a) Reproducibility plots of the best performing FTO|CoZr system with the (inset) OER LSV polarization curves indicating the consistency in the OER onset potential and the overpotential at a current density of 10 mA cm^{-2}. (b) Example of O_2 evolution on a FTO|CoZr sample and corresponding current trace. Chronoamperometry is performed for 4 h at 1.6 V vs. RHE in a 1 M KOH aqueous solution, without initial CV activation, with the grey areas indicating the O_2 baseline measurements. (c) Photograph of a typical FTO|CoZr working electrode in operation.

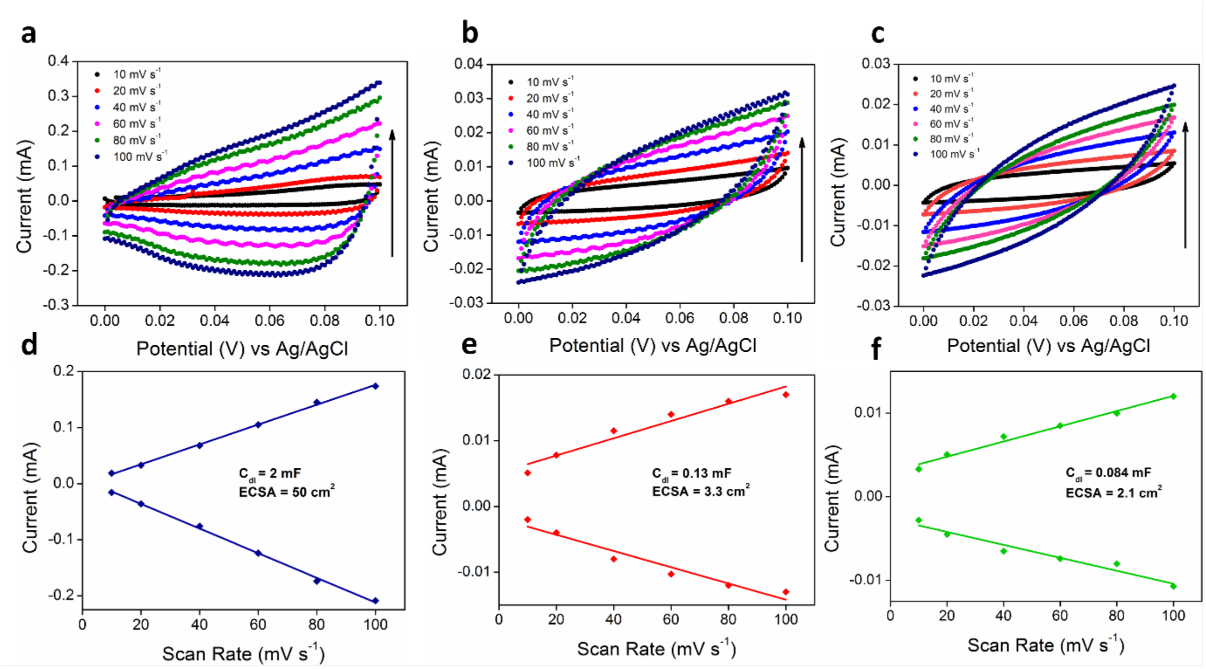

Figure S19. Electrochemically active surface area measurements. Top panel: CV plots of the (a) CoZr, (b) FeZr and (c) CuZr systems recorded at different scan rates. Bottom panel: Plot of current (recorded at a fixed potential) as a function of scan rate for (d) CoZr, (e) FeZr and (f) CuZr systems.

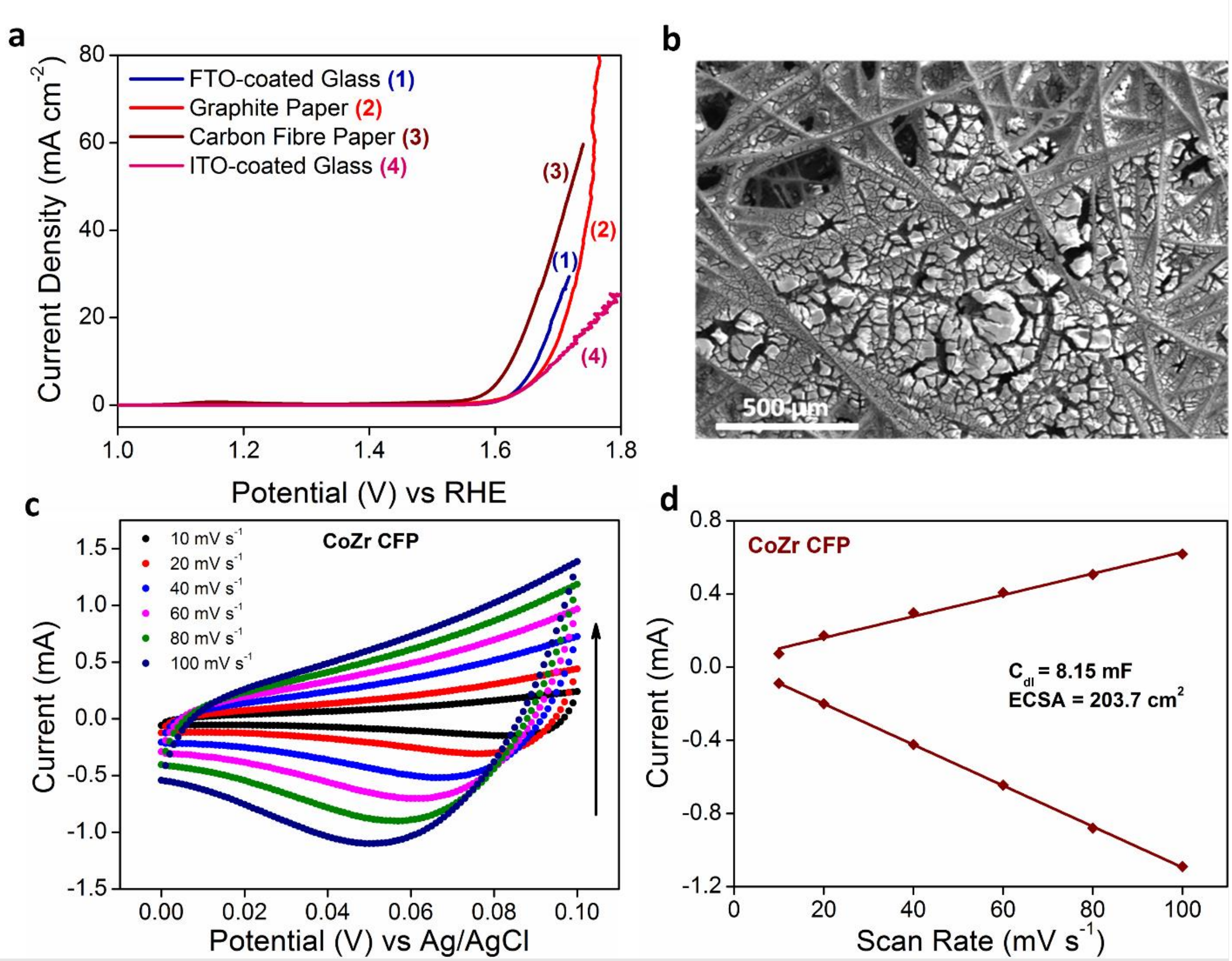

Figure S20. Substrate comparison experiments for OER: (a) LSV polarization curves recorded at a scan rate of 5 mV s^{-1} for the best performing CoZr system on different substrates in 1M KOH (pH 14) at 25˚C. The overpotentials (vs. RHE) at 10 mA cm^{-2} are estimated to be: ~430 mV (on FTO), ~453 mV (on graphite foil), ~390 mV (on carbon-fibre paper) and ~469 mV (on ITO). (b) SEM image of CoZr drop-casted on carbon-fiber paper. (c) ECSA measurements: CV plots of CoZr deposited on carbon-fiber paper recorded at different scan rates. (d) Plot of current (recorded at fixed potential) as a function of scan rate for CoZr on carbon-fiber paper.

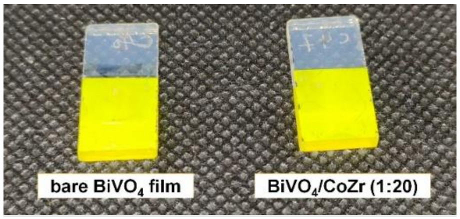

Figure S21. Images of (left) a bare BiVO4 film and (right) a $BiVO_4$ film over which CoZr (1:20) has been spin-coated. The images reveal that the deposition of CoZr is uniform and does not occlude light transmission.

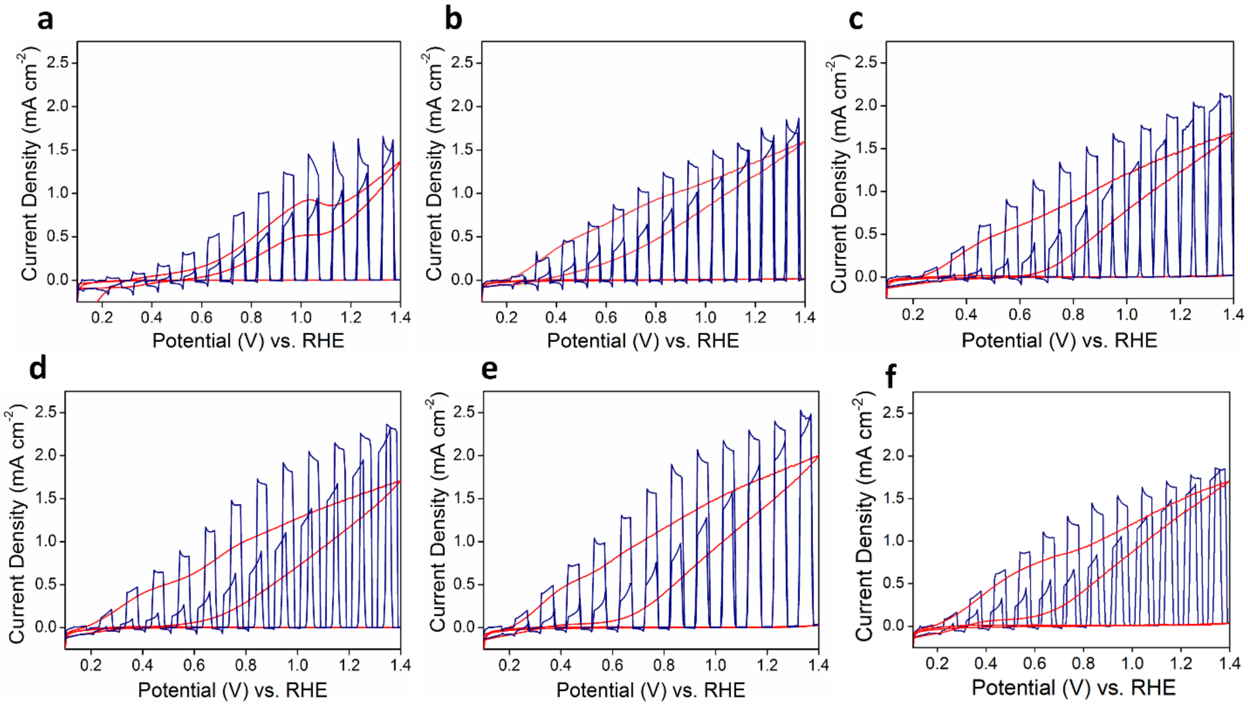

Figure S22. PEC responses of FTO|$BiVO_4$|CoZr system recorded in 0.1 M KBi, 0.1 M K_2SO_4 (pH 8.5) buffer solution at 25˚C without stirring. CV scans under chopped, continuous and no artificial sunlight irradiation (AM 1.5G, 1000 W m^{-2}) of the photoanode with **(a)** no CoZr co-catalyst, **(b)** 1:2 diluted CoZr, **(c)** 1:5 diluted CoZr, **(d)** 1:10 diluted CoZr, **(e)** 1:20 diluted CoZr and **(f)** 1:40 diluted CoZr spin-coated over the $BiVO_4$ layer. The wave at approximately 1V vs. RHE in (a) is owing to the absence of stirring. [S3]

Table S11. Summary of active metal content (from ICP-OES), onset potential, Tafel slope and duration of stability for the three electrocatalytic systems.

System	Metal Content (*10^{-5} mol cm^{-2})	Potential (V) vs. RHE at j =1 mA cm^2	Tafel Slope (mV dec^{-1})	TOF (s^{-1}) (at overpotential: 490 mV vs. RHE)	Stability (h)
CoZr	0.04 (Co)	1.6	54	0.18	18
FeZr	0.05 (Fe)	1.9	75	0.0005	18
CuZr	0.05 (Cu)	1.92	70	0.0002	18

Table S12. Comparison of synthesis procedures and performance metrics of literature bimetallic and trimetallic catalysts with the materials reported in this work.

**Material**	**Substrate**	**Fabrication**	**Onset Potential (V vs. RHE)**	**Overpotential (mV vs. RHE)**	**Tafel slope (mV dec^{-1})**	**Ref.**
CoFeZr nanosheet	Ni foam	Hydrothermal (160ºC; 15h)	~1.46*	264 @20 mA cm^{-2}	54.2	S4
Co/ZrP	RDE	solution route	~1.61*	451 @3 mA cm^{-2}	79	S5
Fe/Co-MOF	FTO	Hydrothermal (120ºC; 24 h)	~1.55*	410 @10 mA cm^{-2}	101	S6
Co(Ox)P@PNC	Si & Cu foil	PLD+NP deposition	1.5	349 @10 mA cm^{-2}	75.3	S7
$Co_{0.89}Ca_{0.11}$-CP	GC	solution route	~1.55*	-	58.3	S8
CoFeOx	FTO	electrodeposition	~1.51*	240 @10 mA cm^{-2}	-	S9
Amorphous Co Phylosilicate	CFP	Hydrothermal+ annealing (200ºC; 24h)	~1.53*	367 @10 mA cm^{-2}	60	S10
$Co_{2-x}V_xO_4$	GC	solution route	-	240 @10 mA cm^{-2}	45	S11
$FeCo_2O_4$	GC	MW-assisted solvothermal	1.57	-	83.2	S12
3DOM Ce-$LaCoO_3$-0.05	RDE	Multi-step solution route + calcination	~1.56*	440 @10 mA cm^{-2}	83	S13
CoZr	FTO	Drop-cast SSP solution	1.56	372 @1 mA cm^{-2} 430 @10 mA cm^{-2}	53	This work
FeZr	FTO	Drop-cast SSP solution	1.77	670 @1 mA cm^{-2}	362	This work
CuZr	FTO	Drop-cast SSP solution	1.81	693 @1 mA cm^{-2}	478	This work

* *Onset potentials estimated from the LSV curves.*

RDE: rotating disk electrode; FTO: fluorine-doped tin oxide; GC: glassy carbon; CFP: carbon fibre paper; ITO: indium tin oxide; PLD: pulsed-laser deposition; MW: microwave.

References:

[S1] S. Eslava, B. P. R. Goodwill, M. McPartlin, D. S. Wright, *Inorg. Chem.* 2011, *50*, 5655.

[S2] D. J. Miller, M. C. Biesinger, N. S. McIntyre, *Surf. Interface Anal.* 2002, *33*, 299.

[S3] V. Andrei, R. L. Z. Hoye, M. Crespo-Quesada, M. Bajada, S. Ahmad, M. D. Volder, R. Friend, E. Reisner, *Advanced Energy Materials* 2018, *8*, 1801403.

[S4] Huang, L.; Chen, D.; Luo, G.; Lu, Y.; Chen, C.; Zou, Y.; Dong, C.; Li, Y.; Wang, S. Zirconium-Regulation-Induced Bifunctionality in 3D Cobalt–Iron Oxide Nanosheets for Overall Water Splitting. *Adv. Mater.* 2019, *31* (28), 1901439.

[S5] Ramos-Garcés, M. V.; Sanchez, J.; La Luz-Rivera, K.; Del Toro-Pedrosa, D. E.; Jaramillo, T. F.; Colón, J. L. Morphology Control of Metal-Modified Zirconium Phosphate Support Structures for the Oxygen Evolution Reaction. *Dalton Trans.* 2020, *49* (12), 3892–3900.

[S6] Iqbal, B.; Saleem, M.; Arshad, S. N.; Rashid, J.; Hussain, N.; Zaheer, M. One-Pot Synthesis of Heterobimetallic Metal–Organic Frameworks (MOFs) for Multifunctional Catalysis. *Chem. Eur. J.* 2019, *25* (44), 10490–10498.

[S7] Bayatsarmadi, B.; Zheng, Y.; Casari, C. S.; Russo, V.; Qiao, S.-Z. Pulsed Laser Deposition of Porous N-Carbon Supported Cobalt (Oxide) Thin Films for Highly Efficient Oxygen Evolution. *Chem. Commun.* 2016, *52* (80), 11947–11950.

[S8] Su, P.; Ma, S.; Huang, W.; Boyjoo, Y.; Bai, S.; Liu, J. Ca^{2+} -Doped Ultrathin Cobalt Hydroxyl Oxides Derived from Coordination Polymers as Efficient Electrocatalysts for the Oxidation of Water. *J. Mater. Chem. A* 2019, *7* (33), 19415–19422.

[S9] Morales-Guio, C. G.; Liardet, L.; Hu, X. Oxidatively Electrodeposited Thin-Film Transition Metal (Oxy)Hydroxides as Oxygen Evolution Catalysts. *J. Am. Chem. Soc.* 2016, *138* (28), 8946–8957.

[S10] Kim, J. S.; Park, I.; Jeong, E.-S.; Jin, K.; Seong, W. M.; Yoon, G.; Kim, H.; Kim, B.; Nam, K. T.; Kang, K. Amorphous Cobalt Phyllosilicate with Layered Crystalline Motifs as Water Oxidation Catalyst. *Adv. Mater.* 2017, *29* (21), 1606893.

[S11] Jiang, C.; Yang, J.; Han, X.; Qi, H.; Su, M.; Zhao, D.; Kang, L.; Liu, X.; Ye, J.; Li, J.; Guo, Z.-X.; Kaltsoyannis, N.; Wang, A.; Tang, J. Crystallinity-Modulated $Co_{2-x}V_xO_4$ Nanoplates for Efficient Electrochemical Water Oxidation. *ACS Catal.* **2021**, *11* (24), 14884–14891.

[S12] Harada, M.; Kotegawa, F.; Kuwa, M. Structural Changes of Spinel MCo_2O_4 (M = Mn, Fe, Co, Ni, and Zn) Electrocatalysts during the Oxygen Evolution Reaction Investigated by In Situ X-Ray Absorption Spectroscopy. *ACS Appl. Energy Mater.* **2022**, *5* (1), 278–294.

[S13] Boonlha, S.; Chakthranont, P.; Kityakarn, S. 3DOM Cerium Doped $LaCoO_3$ Bifunctional Electrocatalysts for the Oxygen Evolution and Reduction Reactions. *ChemCatChem* **2022**, *14* (3).
